# Self-care dependency assessment of person with lower limb amputation: an exploratory study

**DOI:** 10.1590/1518-8345.7424.4332

**Published:** 2024-10-25

**Authors:** Diana Fonseca Rodrigues, Paulo Alexandre Puga Machado, Teresa Martins, António Luís Rodrigues Faria de Carvalho, Cristina Maria Correia Barroso Pinto

**Affiliations:** ^1^ Center for Health Technology and Services Research, Porto, Portugal.; ^2^ University of Porto, School of Medicine and Biomedical Sciences, Porto, Portugal.; ^3^ Nursing School of Porto, Porto, Portugal.; ^4^ Scholarship holder at the Foundation for Science and Technology (FCT), Portugal.

**Keywords:** Activities of Daily Living, Amputation, Disabled Person, Lower Limb, Self-care, Vascular Disease

## Abstract

to identify the sociodemographic and clinical characteristics of the person with dysvascular major lower limb amputation and to assess their degree of dependence and autonomy in self-care activities at home.

quantitative, exploratory, cross-sectional and descriptive study. The convenience sample consisted of 40 participants. A sociodemographic questionnaire and the short version of the Self-Care Dependence Assessment Form were used for data collection.

of the 40 (100%) participants, the majority were male; 75% were over 65 years of age, 77.5% had a transfemoral amputation, and 72.5% were confined to a wheelchair. The higher levels of dependency predominated in self-care: “walking”, “bathing”, “dressing and undressing”, “using the toilet” and “transferring”.

this study showed that the self-care domain with the highest level of dependence is “walking” self-care, and the lowest is “feeding”. Greater autonomy in using the toilet, walking and transferring from bed to chair were shown to be self-care activities with the best ability to predict patient autonomy.

## Highlights

**(1)** Dysvascular major lower limb amputation interferes with activities of daily living.

**(2)** Self-care with the highest level of dependency is “walking”.

**(3)** Self-care with the lowest level of dependency is “feeding”.

**(4)** Develop future interventions on the degree of dependency of patients with dysvascular amputation.

## Introduction

 Lower limb amputation (LLA) changes and affects several aspects of the amputee’s life and poses challenges for them. Lower limb amputation (LLA) has a global and significant impact on the morbidity of amputees ^(^ 1 ^)^ . The most common cause of lower limb amputation is dysvascular amputation, defined as secondary to the complications of peripheral arterial disease, diabetes mellitus, or both, with more than 80% of lower limb amputations due to dysvascular etiology ^(^ 2 ^-^ 3 ^)^ . Peripheral arterial disease (PAD) occurs in people with diabetes. It is often asymptomatic, with an estimated prevalence of 10% to 20%. In addition, the presence of diabetes leads to chronic inflammation and oxidative stress, which further exacerbate PAD and delay the body’s ability to repair ischemic tissue. The presence of these pathological processes can lead to a greater incidence of complications, including pain, reduced functionality and increased risk of death ^(^ 4 ^)^ . 

 A lower limb amputation is a life-changing event that can have a negative impact on a person’s physical and mental health ^(^ 2 ^)^ . Only two-thirds of patients are referred to limb-fitting centers after amputation, and only 40% of these patients ultimately receive a prosthetic limb. As a result, most patients remain wheelchair dependent after amputation ^(^ 5 ^-^ 6 ^)^ . Functional capacity 12 months after dysvascular LLA reported poor physical function in observational studies, with only 39% returning to previous levels of mobility ^(^ 7 ^)^ . Rehabilitation outcomes following dysvascular amputation are poor, with patients experiencing greater disability than 95% of the general population ^(^ 8 ^)^ . 

 People with peripheral artery disease (PAD) and diabetes who undergo amputation often have pre-existing health problems and may experience cognitive impairment ^(^ 9 ^)^ . Dysvascular limb loss is associated with a high prevalence of multiple health problems that can adversely affect an individual’s overall well-being and functional ability ^(^ 10 ^)^ . Dysvascular LLAs undergoing transfemoral amputation have a higher risk of mortality within the first year after surgery, indicating the presence of more severe vascular disease. The frailty of this population is reflected in a higher mortality rate in most elderly patients ^(^ 11 ^)^ . 

 Mortality is particularly high in patients undergoing transfemoral amputation within the first year after surgery. The higher mortality rates observed in the elderly, especially those with severe cardiac disease and undergoing hemodialysis, indicate the vulnerability of this population. Dysvascular amputation of the lower limb has emerged as a major contributor to the global prevalence of disability ^(^ 11 ^-^ 12 ^)^ . People with dysvascular lower limb amputations face challenges in engaging in physical activity due to chronic disease, severe disability, and unaddressed psychological and social factors ^(^ 13 ^)^ . Lower limb (LL) amputees with dysvascular conditions report a decline in their functional abilities, leading to a perception of reduced physical ability and independence ^(^ 14 ^)^ . 

 The LLA is responsible for physical disabilities that can limit an amputee’s ability to function in everyday life. Patients may experience debilitating loss of independence, which can ultimately lead to physical, behavioral, and psychological changes. Mobility is altered in all amputees, physical activity is reduced, and it is difficult for patients to remain active due to the increased energy expenditure ^(^ 1 ^)^ . Lower limb amputees face a fundamental functional problem, and the limitation of independent movement in daily life can lead to increased dependency ^(^ 1 ^)^ . 

 A dependent person is defined as one who has a limited capacity or inability to initiate and develop activities important for well-being, health, and maintenance of life without the assistance of another person ^(^ 15 ^)^ . This includes activities of daily living such as bathing, personal hygiene, transferring, using the toilet, walking, feeding, and positioning ^(^ 16 ^)^ . To understand a person’s true needs, it is not enough to say that the person is dependent on others for self-care; the type, amount and nature of support required may vary depending on the person’s area of dependency and the skills of the caregiver ^(^ 17 ^)^ . The use of a robust and reliable assessment tool that can evaluate the level of dependency according to different areas of self-care is very important for healthcare professionals to be able to design a discharge plan that is patient-centered ^(^ 17 ^)^ . 

This study is part of a larger doctoral research project that identifies and recognizes the profile of the person with dysvascular major lower limb amputation. The objectives of this study were: to identify the sociodemographic and clinical characteristics of the person with dysvascular major lower limb amputation and to assess the degree of dependence and autonomy in self-care activities at home.

This information can contribute to the development of interventions and programs to empower amputees and caregivers regarding the reliance on self-care associated with the disability of a dysvascular major lower limb amputation in the transition to home.

## Method

### Study design and settings

This was an exploratory, cross-sectional, descriptive study of a quantitative nature, conducted according to the Strengthening the Reporting of Observational Studies in Epidemiology (STROBE) guidelines for observational studies, with the aim of assessing the dependence on self-care in activities of daily living of the person with dysvascular major lower limb amputation. This study is part of a larger exploratory, cross-sectional, mixed-methods study with a predominantly qualitative paradigm that is part of a PhD project in nursing. The study was conducted in a hemodynamic consultation for the follow-up of vascular diseases in the vascular surgery service of a hospital unit in northern Portugal. Data collection took place between May 2022 and June 2023.

### Population and sample

The sample population of this study was selected from patients attending the hemodynamic consultation for vascular disease in a vascular surgery service in a hospital in northern Portugal, where the study was conducted. Patients with dysvascular major lower limb amputation who attended the consultation were recruited into the study according to the following inclusion criteria: (1) amputees aged 18 years or older, (2) major lower limb amputation due to vascular etiology, (3) living at home, (4) receiving assistance with activities of daily living (ADLs) at home from a family caregiver, and (5) cognitive ability to understand. Exclusion criteria were as follows: (1) refusal to sign the informed consent form, (2) independence in all ADLs, and (3) living in nursing homes and institutions.

Convenience sampling was used and a total of 40 patients with dysvascular major lower limb amputation were recruited for the study. All study participants were living at home at the time of data collection. Data collection was planned for a period of one year, with a total of 13 months of data collection to contact the entire hospitalizable population with major lower limb amputation due to PAD who were being followed in the clinic. New patients without an appointment were identified and included in the study. Of the accessible population, five amputees died, five refused to participate, seven did not meet the inclusion criteria, and eight missed their appointments and did not reschedule.

### Data collection instruments

The study used two instruments:

a) Questionnaire on socio-demographic and clinical variables: This instrument was developed for this study and consists of two parts, one related to the socio-demographic variables, sex, age, educational level, employment status at the time of surgery, household and destination after discharge, and who support after discharge. Regarding the clinical variables included in the instrument, clinical background, date of surgery, amputation level, discharge date and previous contralateral amputation.

 b) Self-Care Dependency Evaluation Form (SCDEF) Short Version ^(^ 18 ^)^ : This form determines the self-care abilities of patients with dependencies. The instrument consists of 27 items that report self-care assessment activities assigned to over 10 self-care domains: walking, transferring, turning, lifting, using the toilet, feeding, getting ready, dressing and undressing, bathing, and taking medication. Each self-care activity is scored on a four-point Likert scale: (1) dependent, not participating, (2) needs help from another person, (3) needs assistive devices, and (4) completely independent, allowing for an overall assessment of self-care dependency by domain and activity. This instrument has been widely used in the assessment of self-care impairment and has demonstrated good metric properties ^(^ 18 ^)^ . 

### Ethical considerations

The deontological premises recommended by the ethics applied to research with human subjects were followed during the methodological process, the study was approved by the ethics committee of the hospital where the data collection took place, according to the Declaration of Helsinki. Each form was coded with a number to ensure confidentiality. Participants who agreed to participate in the study signed an informed consent form.

### Data analysis

Statistical analyses were performed using IBM SPSS statistical software (version 29). Continuous variables are expressed as mean ± standard deviation, and categorical variables are expressed as percentages or number of observations. A two-step cluster analysis was performed to identify distinct groups within the sample based on levels of self-care dependency. The merging process was based on Schwarz’s Bayesian Criterion (SBC), which helped determine the optimal cluster structure. The quality and validity of the clusters were assessed using silhouette scores ranging from -1 to 1. Higher silhouette values indicate better defined clusters. In addition, SBC values were analyzed to confirm the optimal number of clusters. The resulting clusters were interpreted by analyzing the means and distributions of the self-care dependency scores within each cluster.

## Results

### Sociodemographic and clinical characteristics of the participants

 The study sample consisted of 40 individuals with dysvascular major lower limb amputation; most of the individuals were men (80%) and 20% were women. The age distribution was interval, with the most common age group being between 76 and 80 years (27.5%), and the less common age groups being between 46 and 50 years (2.5%) and 51 and 50 years (2.5%). Regarding the level of education, 70% had four years of basic education, while only 2.5% had a university education. A total of 21% of amputees were retired at the time of surgery and 5.0% were employed. In terms of living situation, 70% of the amputees lived in a household with another person and 5% lived alone. After amputation surgery, 85% of the individuals returned home after hospital discharge, and 24% received support from a spouse or partner after returning home with an amputation. The sociodemographic characteristics of the participants are shown in Table 1 . 

 In Table 2 , we present the clinical characterization of the 40 amputees included in the study; all individuals had peripheral arterial disease (PAD), representing 100% of the sample, and 57.5% also had diabetes mellitus associated with PAD. Regarding the number of comorbidities, 32.5% of the individuals had 3 comorbidities, 32.5% had 2 comorbidities, and only 12.5% had 1 comorbidity (PAD). A total of 77.5% of the amputees had an amputation above the knee, 22.5% had an amputation below the knee, 7.5% had a previous contralateral finger amputation, and 7.5% had a previous contralateral transmetatarsal amputation. At the time of the study, 15% of the participants had been amputees for less than one year, 50% had been amputees for one to five years, and 14% had been amputees for more than five years. In terms of assistive devices used for mobility, 72.5% of the amputees used only a wheelchair for mobility, 10% had a prosthesis but continued to use a wheelchair for mobility, 7.5% had a prosthesis but needed to use crutches, 5% had a prosthesis, and another 5% used a wheelchair and crutches for mobility. 

### Degree of dependency per self-care domain

The assessment of LLA dependency in self-care was conducted according to the 10 domains included in the short version of the SCDEF, with respect to the self-care domain of “walking”, “transferring”, “turning”, “lifting”, “using the toilet”, “feeding”, “getting ready”, “dressing and undressing”, “bathing”, and “taking medication”. Self-care is rated using a four-point Likert scale in each self-care domain: (1) dependent not participating, (2) needs help from another person, (3) needs assistive devices, and (4) completely independent, which helps to assess the level of dependency and needs of the LL amputee regarding activities of daily living.

**Table 1:** - Sociodemographic characteristics of the participants (n = 40). Vila Nova de Gaia, VNG, Portugal, 2022-2023

Variables	Participants (n*)	Percentage (%)
**Gender**		
Male	32	80
Female	8	20
**Age (years)**		
46-50	1	2.5
51-55	1	2.5
56-60	3	7.5
61-65	6	15.0
66-70	9	22.5
71-75	4	10.0
75-80	11	27.5
> 81	5	12.5
**Education level**		
Not formally educated	1	2.5
4 years (1st stage Primary Education)	28	70.0
6 years (2nd stage Primary Education)	3	7.5
9 years (3rd stage Primary Education)	3	7.5
12 years (Secondary Education)	4	10.0
Graduate	1	2.5
**Employment status at time of surgery**		
Active employee	2	5.0
Unemployed	1	2.5
Retired	21	52.5
Early retired	16	40.0
**Household**		
Alone	2	5.0
Lives with one person	28	70.0
Lives with two or more people	10	25.0
**Destination after discharge**		
Home	34	85.0
Sister’s home	1	2.5
Short term inpatient rehabilitation	4	10.0
Daughter’s home	1	2.5
**Who gives support after discharge**		
Friend/Neighbor	1	2.5
Offspring	11	27.5
Spouse/partner	24	60.0
Brother/sister	4	10.0

* n = Number of participants

**Table 2:** - Clinical characteristics of the participants (n = 40). Vila Nova de Gaia, VNG, Portugal, 2022-2023

Variables	Participants (n*)	Percentage (%)
**Clinical background**		
Peripheral Arterial Disease	40	100.0
Dyslipidemia	2	5.0
Hypertension	20	50.0
Smoker	7	17.5
Chronic Obstructive Pulmonary Disease	7	17.5
Heart Disease	7	17.5
Kidney Failure	7	17.5
Diabetes	18	45.0
Diabetic retinopathy	1	2.5
Chronic anemia	1	2.5
Respiratory insufficiency	1	2.5
Hyperactive bladder	1	2.5
Rheumatoid arthritis	1	2.5
**Total comorbidities**		
One comorbidity	5	12.5
Two comorbidities	13	32.5
Three comorbidities	13	32.5
Four comorbidities	6	15.0
Five comorbidities	3	7.5
**Amputation level**		
Transfemoral amputation	31	77.5
Transtibial amputation	9	22.5
**Previous contralateral amputation**		
Absent	34	85.0
Finger amputation	3	7.5
Transmetatarsic amputation	3	7.5
**How long had the amputation**		
< 1 ano	6	15.0
1-5 anos	20	50.0
> 5 anos	14	35.0
**Assistive devices for mobility**		
Wheelchair (only)	29	72.5
Wheelchair and crutches	2	5.0
Prosthesis and wheelchair	4	10.0
Prosthesis and crutches	3	7.5
Prosthesis (only)	2	5.0

* n = Number of participants

Regarding self-care “walking”, three activities are included in this domain to evaluate self-care, keeping the body in an upright position, only 5% of LL amputees can stand up without any kind of help from assistive devices or another person, 70% need to use assistive devices that include crutches or prosthesis, 12.5% can’t stand up. Regarding the activity of walking up and down stairs, 70% of LL amputees are unable to do it, and 22.5% climb up and down stairs using crutches and prosthesis, 7.5% can climb up and down stairs with the help of a person. In the activity of walking medium distances, 72.5% of the LL amputees can do it with the help of crutches, prosthesis or wheelchair; 7.5% of the participants in the study were unable to walk distances even if they had help or assistive devices.

In the self-care “Transferring”, which assesses the ability of LL amputees to transfer from bed to chair/armchair and from chair/armchair to bed, 62.5% require the use of assistive devices to perform the transfer, 27.5% are unable to perform the transfer independently or with the use of assistive devices and require the assistance of another person to perform the transfer. Only 10% of the LL amputees in the study were able to transfer from bed to chair/armchair and from chair/armchair to bed independently, without the use of assistive devices or help from another person.

Regarding the self-care domain “turning”, which assesses whether the person with a lower limb amputation moves the body by moving from one side to the other, the results show that 65% of the amputees need assistive devices, 7.5% are able to turn around only with the help of another person, and 27.5% are completely independent in this domain. Regarding self-care “lifting”, which refers to the ability to lift a part of the body, most of the participants 52.5% of the amputees are independent, 40.0% need assistive devices and 7.5% need help from another person.

In self-care “using the toilet”, regarding the activity positions in toilet or bedpan, 77.5% of LL amputees need assistive devices, in the ability of lifting from toilet 72.5% of participants need assistive devices, regarding the activity arranging clothes after personal hygiene, 27.5% need assistive devices and 45% are completely independent.

Regarding self-care “feeding”, most of the LL amputees 92.5% are independent in opening containers, 97.5% are independent in holding a glass or cup, but 87.5% need help from another person to prepare food to eat.

Nail care is the activity related to self-care “getting ready” with higher dependency with 97.5% needing help from another person, regarding the activities: combing hair, applying deodorant and maintaining oral hygiene, most participants are independent.

Self-care “dressing and undressing” includes five self-care activities, 67.5% of LL amputees need help from another person to tie their shoes and 65% need help from another person to put on socks. A total of 82.5% were completely independent in dressing themselves, 52.5% needed help from another person to choose clothes, and 47.5% needed help from another person to dress the lower part of the body.

One of the self-care activities with a higher level of dependency in the person with LLA is the self-care activity “bathing”, related to obtaining items for bathing, 92.5% of the LL amputees in the study need help from another person, in the ability to wash the body a total of 85.0% need help from another person. A total of 55% of the LL amputees were completely independent in opening the tap for bathing. Regarding the self-care activity “taking medication”, 85.0% of the LL amputees need help from another person to prepare medication, and regarding the activity “taking medication”, 82.5% are independent.

 To create an overall variable, the items within each domain were combined. Table 3 shows the conversion of the ten calculated variables associated with different self-care activities into a single composite variable called the overall dependency level. 

 According to the results presented in Table 4 , the self-care domain with a high level of dependency is self-care “walking” with a mean of 2.28 and a standard deviation of 0.59, followed by self-care “bathing” with a mean of 2. 41 and a standard deviation of 0.47; self-care “dressing and undressing” with a mean of 2.81 and a standard deviation of 0.61%; self-care “using the toilet” with a mean of 2.83 and a standard deviation of 0.65 and self-care “transferring” with a mean of 2.83 and a standard deviation of 0.56%. 

Considering the self-care domains with lower levels of dependency, the self-care “feeding” as the lower level of dependency with a mean of 3.45% and a standard deviation of 0.32%, followed by self-care “lifting” with a mean of 3.45% and a standard deviation of 0. 64%; “dressing and undressing” self-care with a mean of 3.37% and a standard deviation of 0.48%; “turning” self-care with a mean of 3.20% and a standard deviation of 0.56%; and “taking medication” self-care with a mean of 3.19% and a standard deviation of 0.75%.

The global self-care dependency level of the LL amputees in this study presented a mean of 2.99% with a standard deviation of 0.43%, and almost every participant required assistance in the self-care domains assessed. The types of assistive devices used by LL amputees to assist with self-care activities included ambulatory devices such as wheelchairs, crutches, and prosthesis. The LL amputees used shower stools, grab bars, and shower chairs as assistive devices to help in the toilet. In the bedroom, they used the headboard and bed edges to assist with turning, lifting, and transferring, and did not use any assistive devices to adapt to the bed and assist with self-care activities.

table 3

**Table 3:** - Mean, standard deviation, and minimum and maximum for each self-care domain. Vila Nova de Gaia, VNG, Portugal, 2022-2023

Self-care domains	n*	Minimum	Maximum	Mean	Standard deviation
Walking	40	1.00	3.33	2.28	0.59
Transferring	40	2.00	4.00	2.83	0.59
Turning	40	2.00	4.00	3.20	0.56
Lifting	40	2.00	4.00	3.45	0.64
Using the toilet	40	1.00	3.33	2.83	0.65
Feeding	40	1.75	4.00	3.45	0.32
Getting ready	40	1.25	3.50	3.37	0.48
Dressing and undressing	40	1.20	3.80	2.81	0.61
Bathing	40	1.00	3.33	2.41	0.47
Taking medication	40	1.50	4.00	3.19	0.75
Overall level of dependency	40	1.38	3.52	2.99	0.43

* n = Number of participants in the study

 Two-step cluster analysis identified two distinct clusters within the sample of individuals with dysvascular major lower limb amputation, with high silhouette scores indicating good cohesion and separation between the clusters. Cluster 1 included a higher proportion of older, frail and dependent patients. Table 4 provides a detailed description of the clusters formed, including their sociodemographic and clinical characteristics, as well as their level of dependence on self-care. 

**Figure 1:**
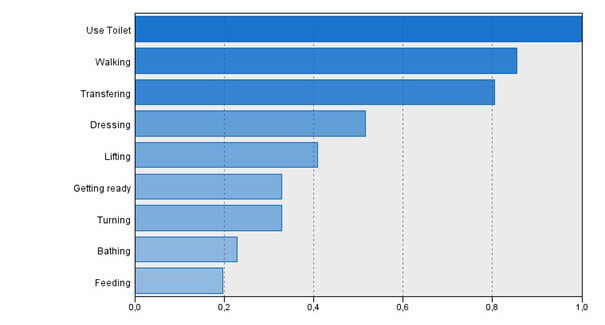
- Cluster predictors

 Greater autonomy in using the toilet, walking, and transferring from bed to chair was shown to be self-care activities with the best ability to predict the autonomy of this type of patient ( Figure 1 ). 

## Discussion

To the best of our knowledge, this study is the first to examine self-care dependency in activities of daily living in individuals who have experienced a dysvascular major lower limb amputation. This study used an instrument that allows for a prioritized and operational assessment of self-care competencies in individuals with major lower limb amputations divided into 10 domains.

 By focusing on individuals who have undergone a dysvascular major lower limb amputation, this study provides valuable insights into their sociodemographic and clinical characteristics. It also examines the extent to which they rely on assistance with activities of daily living. In total, 80% of the sample of 40 were men and 20% were women. Previous studies have reported that men have a higher risk of lower limb amputation than women ^(^ 19 ^)^ , which may be explained by higher rates of peripheral arterial disease, peripheral neuropathy, and smoking in men than in women ^(^ 20 ^-^ 21 ^)^ . The lower incidence of lower limb amputation in women with vascular disease may be explained by the effect of estrogen in reducing vascular pathology ^(^ 22 ^)^ . 

 Regarding the age of the lower limb amputees included in our study, the most common age range was between 76 and 80 years, representing 27.5% of the study sample, and the least common age range was between 46 and 50 years, representing only 2.5% of the participants. Previous evidence suggests that most amputations today are secondary to dysvascular disease, and the prevalence of PAD increases significantly with age in both men and women. In people younger than 50 years, the prevalence is between 15% and 20%, rising to between 15% and 20% by the age of 80 years ^(^ 23 ^-^ 24 ^)^ . 

table 4

**Table 4:** - Cluster characteristics. Vila Nova de Gaia, VNG, Portugal, 2022-2023

	Cluster 1 (n*=9)	Cluster 2 (n*=31)
Male	88.8% (8)	77.4% (24)
Primary school	100% (9)	61.3% (19
Transfemoral amputation	88.8% (8)	74.2% (23)
Walking	M=1.44	M=2.53
Transferring	M=2.00	M=3.06
Using the toilet	M=1.85	M=3.11
Feeding	M=3.22	M=3.52
Getting ready	M=2.92	M=3.50
Dressing and undressing	M=2.09	M=3.01
Lifting	M=2.78	M=3.65
Turning	M=2.67	M=3.35

 In the present study, 40% of dysvascular lower limb amputees were in early retirement prior to amputation. Previous studies have reported that intermittent claudication is a manifestation of peripheral arterial disease, resulting in reduced mobility and quality of life ^(^ 25 ^)^ . Even in patients with peripheral arterial disease with or without atypical symptoms, functional impairment is present; frailty is common in patients with symptomatic PAD and is associated with walking impairment ^(^ 25 ^-^ 26 ^)^ . 

 According to previous findings, older patients are less likely to mobilize with prosthesis than their younger counterparts, because the level of amputation patients with transtibial amputation are twice as likely to mobilize with prosthesis than patients with transfemoral amputation ^(^ 27 ^)^ . In our study sample, 72.5% of the lower limb amputees were over 65 years of age, 77.5% had a transfemoral amputation, and 72.5% were confined to a wheelchair. Authors ^(^ 28 ^)^ reported that in a period of 3 months after amputation, all age groups of lower limb amputees presented the lowest scores of physical functions, and at 12 months after amputation, there is a difference between age groups at a functional level, with an obvious loss of function in the oldest patients. These patients need a special focus that requires daily rehabilitation to regain their basic physical functions. Physical function in lower limb amputees may also be affected by the number of comorbidities present. 

 Our results showed that 65% of the lower limb amputees in our study had at least two or three comorbidities. Adults who have undergone lower-limb amputation due to vascular disease often have multiple health conditions ^(^ 29 ^)^ . The presence of multiple comorbidities in patients with advanced peripheral vascular disease often results in increased levels of frailty ^(^ 30 ^)^ . 

 Poor physical outcomes, including balance problems, decreased strength, and limited mobility or ability to walk, are commonly observed in patients with dysvascular lower limb amputations. People with dysvascular lower limb amputations often experience a range of health problems that affect their physical well-being and ability to perform daily tasks ^(^ 10 ^,^ 31 ^)^ . Our study found that statistically, the self-care domains with higher dependency in dysvascular major lower limb amputees were “walking”, “bathing”, “dressing and undressing”, “using the toilet”, “transferring”, with self-care walking being the one with higher dependency in our participants. Walking and climbing stairs are the most difficult activities for individuals who have undergone a lower-limb amputation. Individuals with transfemoral amputations are particularly affected and are unable to regain their ability to perform basic and instrumental activities of daily living ^(^ 32 ^)^ . 

 With regard to the areas of self-care with higher dependency on ADLs identified in our study, the literature indicates that patients over 12 months post-amputation require more assistance with personal hygiene, bathing, dressing and undressing, and using the toilet ^(^ 28 ^)^ . The most significant decline in mobility, particularly in independent walking, was observed 12 months after amputation. The patient’s ability to walk unassisted depends on the presence of a prosthetic limb ^(^ 28 ^)^ . Of all the participants in our study, only 5% were able to walk using prosthetics alone. Twelve months after amputation, the majority of lower limb amputees are still unable to regain the same level of independence in their daily activities ^(^ 28 ^)^ . 

 Nurses play an important role in encouraging dysvascular lower limb amputees to regain independence in self-care activities. According to our cluster analysis, greater self-care autonomy in using the toilet, walking, and transferring from bed to chair can predict autonomy in this type of patient. Previous studies have shown that independent walking helps regain independence in performing basic skills and activities of daily living ^(^ 33 ^)^ . Home rehabilitation interventions for dysvascular lower limb amputees with exercises to improve standing balance, sitting and standing ability, transfers, and mobility help these amputees regain mobility and functional independence sooner, contributing to improved mobility and quality of life ^(^ 34 ^)^ . 

There are some limitations to this study, such as the small sample size of non-probability patients from a single hospital in northern Portugal. Therefore, caution should be exercised when generalizing the results. To improve future studies, it is recommended to increase the sample size and extend the study duration. Despite certain limitations, this study sheds light on the impact of dysvascular major lower limb amputation on activities of daily living. These findings can provide considerations for planning future research on the dependency of a person with a dysvascular major lower limb amputation in activities of daily living and for developing interventions and programs to help train patients, caregivers, and families.

## Conclusion

This study showed that the self-care domain with the highest level of dependence is “walking” self-care, and the lowest is “feeding”. Greater autonomy in using the toilet, walking and transferring from bed to chair were shown to be self-care activities with the best ability to predict patient autonomy. The patients with this type of amputation are over 65 years of age; only 5% are able to ambulate with prosthesis and have some level of dependency in the self-care domains related to activities of daily living.

Using a reliable assessment tool to evaluate the level of dependency in the different self-care domains of a person with a dysvascular major lower limb amputation, it is important to identify their needs regarding self-care activities. This can help to understand the functional impact and disability that a dysvascular amputation brings, and the level of assistance the person with this type of amputation needs with their activities of daily living.

Today, there is a trend toward early discharge with an early return to the community/home when the amputee is still totally dependent on others. Assessing the dependency of a person with a dysvascular major lower limb amputation in activities of daily living is essential for designing educational interventions and programs to improve their ability to perform basic activities of daily living. Rehabilitation interventions should continue after discharge with exercises to improve functional capacity and independence in self-care activities. Family caregivers should be involved from the beginning of these interventions and programs to assist and support the lower limb amputee in regaining some level of independence in performing activities of daily living, thereby contributing to an improved quality of life.
