# Supplementary material for: Self-care dependency assessment of person with lower limb amputation: an exploratory study
Source: Rev Lat Am Enfermagem. 2024 Oct 25;32:e4332. doi: 10.1590/1518-8345.7424.4332 (PMC11526329; doi:10.1590/1518-8345.7424.4332)
Supplement: Translation

# Translation: Evaluación de la dependencia del autocuidado en personas con amputaciones de los miembros inferiores: un estudio exploratorio

**DOI:** 10.1590/1518-8345.7424.4331

**Published:** 2024-10-02

**Authors:** Diana Fonseca Rodrigues, Paulo Alexandre Puga Machado, Teresa Martins, António Luís Rodrigues Faria de Carvalho, Cristina Maria Correia Barroso Pinto

**Affiliations:** ^1^ Center for Health Technology and Services Research, Porto, Portugal.; ^2^ University of Porto, School of Medicine and Biomedical Sciences, Porto, Portugal.; ^3^ Nursing School of Porto, Porto, Portugal.; ^4^ Becaria de la Foundation for Science and Technology (FCT), Portugal.

**Keywords:** Actividades Cotidianas, Amputación, Persona con Discapacidad, Miembro Inferior, Autocuidado, Enfermedad Vascular

## Abstract

identificar las características sociodemográficas y clínicas de personas con amputación mayor de miembro inferior de origen vascular y evaluar su nivel de dependencia y autonomía en las actividades de autocuidado en el hogar.

estudio cuantitativo, exploratorio, transversal y descriptivo. La muestra por conveniencia estuvo compuesta por 40 participantes. Para la recolección de datos se utilizó un cuestionario sociodemográfico y la versión corta del Formulario de Evaluación de Dependencia de Autocuidado.

de los 40 (100%) participantes, la mayoría eran hombres; el 75% tenía más de 65 años y el 77,5% fue sometido a amputación transfemoral. En relación al nivel de dependencia predominaron las actividades relacionadas con el autocuidado: “caminar”, “bañarse”, “vestirse y desvestirse”, “ir al baño” y “trasladarse” y en cuanto a la autonomía el 72,5 % se limitó a un silla de ruedas.

el dominio del autocuidado con mayor nivel de dependencia es “caminar” y el más bajo es el “alimentarse”. Una mayor autonomía en el uso del baño, caminar y pasar de la cama a la silla resultaron ser actividades de autocuidado con mayor capacidad para predecir la autonomía del paciente.

## Destacados

**(1)** La amputación disvascular mayor del miembro inferior interfiere con las actividades de la vida diaria.

**(2)** El autocuidado con mayor nivel de dependencia es “caminar”.

**(3)** El nivel más bajo de dependencia del autocuidado es “alimentarse”.

**(4)** Desarrollar futuras intervenciones sobre el grado de dependencia de los pacientes con amputación disvascular.

## Introducción

 La amputación de miembros inferiores (AMI) altera y afecta varios aspectos de la vida del amputado y presenta desafíos para él. La AMI tiene un impacto global y significativo en la morbilidad de los amputados ^(^ 1 ^)^ . La causa más común de amputación de extremidad inferior es la amputación disvascular, definida como secundaria a las complicaciones de enfermedad arterial periférica, diabetes mellitus (DM) o ambas, con más del 80% de las amputaciones de extremidad inferior debido a la etiología disvascular ^(^ 2 ^-^ 3 ^)^ . La enfermedad arterial periférica (EAP) ocurre en personas con DM. Generalmente es asintomática, con prevalencia estimada del 10% al 20%. Además, la presencia de DM conduce a inflamación crónica y estrés oxidativo, que exacerban aún más la EAP y retrasan la capacidad del cuerpo para reparar el tejido isquémico. La presencia de estos procesos patológicos puede llevar a una mayor incidencia de complicaciones, incluyendo dolor, reducción de la funcionalidad y aumento del riesgo de muerte ^(^ 4 ^)^ . 

 Una amputación de extremidad inferior es un evento que cambia la vida y puede tener un impacto negativo en la salud física y mental de una persona ^(^ 2 ^)^ . Solo dos tercios de los pacientes son referidos a centros de adaptación de miembros después de la amputación, y solo el 40% de estos pacientes terminan recibiendo una prótesis. Como resultado, la mayoría de los pacientes permanece dependiente de silla de ruedas después de la amputación ^(^ 5 ^-^ 6 ^)^ . La capacidad funcional 12 meses después de la AMI disvascular reportó mala función física en estudios observacionales, con solo el 39% regresando a los niveles anteriores de movilidad ^(^ 7 ^)^ . Los resultados de la rehabilitación después de la amputación disvascular son pobres, con los pacientes presentando mayor incapacidad que el 95% de la población general ^(^ 8 ^)^ . 

 Las personas con EAP y DM que sufren amputación generalmente tienen problemas de salud preexistentes y pueden presentar deterioro cognitivo ^(^ 9 ^)^ . La pérdida de miembros disvasculares está asociada a alta prevalencia de múltiples problemas de salud que pueden afectar negativamente el bienestar general y la capacidad funcional de un individuo ^(^ 10 ^)^ . Las AMI disvasculares sometidas a amputación transfemoral tienen un mayor riesgo de mortalidad en el primer año después de la cirugía, indicando la presencia de enfermedad vascular más grave. La fragilidad de esta población se refleja en una tasa de mortalidad más alta en la mayoría de los pacientes ancianos ^(^ 11 ^)^ . 

 La mortalidad es particularmente alta en pacientes sometidos a amputación transfemoral en el primer año después de la cirugía. Las tasas de mortalidad más altas observadas en ancianos, especialmente aquellos con enfermedad cardíaca grave y sometidos a hemodiálisis, indican la vulnerabilidad de esta población. La amputación disvascular de miembro inferior surgió como uno de los principales contribuyentes a la prevalencia global de discapacidad ^(^ 11 ^-^ 12 ^)^ . Las personas con amputaciones disvasculares de miembros inferiores enfrentan desafíos para participar en actividades físicas debido a enfermedades crónicas, discapacidad grave y factores psicológicos y sociales no abordados ^(^ 13 ^)^ . Los amputados de miembros inferiores (MI) con condiciones disvasculares reportan un declive en sus habilidades funcionales, llevando a una percepción de capacidad física e independencia reducidas ^(^ 14 ^)^ . 

 La AMI es responsable de discapacidades físicas que pueden limitar la capacidad de un amputado para realizar actividades en la vida diaria. Los pacientes pueden sufrir una pérdida debilitante de independencia, que puede llevar a cambios físicos, conductuales y psicológicos. La movilidad se altera en todos los amputados, la actividad física se reduce y es difícil para los pacientes permanecer activos debido al aumento del gasto energético ^(^ 1 ^)^ . Los amputados de miembros inferiores enfrentan un problema funcional fundamental, y la limitación del movimiento independiente en la vida diaria puede llevar al aumento de la dependencia ^(^ 1 ^)^ . 

 Una persona dependiente se define como aquella que tiene una capacidad limitada o incapacidad para iniciar y desarrollar actividades importantes para el bienestar, la salud y el mantenimiento de la vida sin la asistencia de otra persona ^(^ 15 ^)^ . Esto incluye actividades de la vida diaria, como bañarse, higiene personal, transferencia, ir al baño, caminar, alimentarse y posicionarse ^(^ 16 ^)^ . Para entender las verdaderas necesidades de una persona, no basta con decir que es dependiente de otros para el autocuidado; el tipo, la cantidad y la naturaleza del apoyo necesario pueden variar dependiendo del área de dependencia de la persona y las habilidades del cuidador ^(^ 17 ^)^ . El uso de una herramienta de evaluación robusta y confiable que pueda evaluar el nivel de dependencia según las diferentes áreas de autocuidado es muy importante para que los profesionales de la salud puedan elaborar un plan de alta centrado en el paciente ^(^ 17 ^)^ . 

Este estudio forma parte de un proyecto de investigación doctoral más amplio que identifica y reconoce el perfil de la persona con amputación de miembro inferior mayor disvascular. Los objetivos de este estudio fueron: identificar las características sociodemográficas y clínicas de personas con amputación mayor de miembro inferior de origen vascular y evaluar su nivel de dependencia y autonomía en las actividades de autocuidado en el hogar.

Esta información puede contribuir al desarrollo de intervenciones y programas para capacitar a los amputados y cuidadores en relación con la dependencia del autocuidado asociado a la discapacidad de una amputación mayor de miembro inferior por enfermedad arterial periférica en la transición al hogar.

## Métodos

### Diseño y localización del estudio

 Este fue un estudio exploratorio, transversal y descriptivo de naturaleza cuantitativa, llevado a cabo según las directrices del *Strengthening the Reporting of Observational Studies in Epidemiology* (STROBE) para estudios observacionales, con el objetivo de evaluar la dependencia del autocuidado en las actividades de vida diaria de la persona con amputación mayor de miembro inferior de origen vascular. Este estudio forma parte de un estudio más amplio, exploratorio, transversal y de métodos mixtos, con un paradigma predominantemente cualitativo, que forma parte de un proyecto de doctorado en enfermería. El estudio se realizó en una consulta de hemodinámica para el seguimiento de enfermedades vasculares en el servicio de cirugía vascular de una unidad hospitalaria del norte de Portugal. La recolección de datos ocurrió entre mayo de 2022 y junio de 2023. 

### Población y muestra

La población de muestra de este estudio fue seleccionada entre los pacientes que asistieron a la consulta de hemodinámica para enfermedad vascular en un servicio de cirugía vascular en un hospital en el norte de Portugal, donde se realizó el estudio. Los pacientes con amputación mayor de miembro inferior disvascular que asistieron a la consulta fueron reclutados para el estudio según los siguientes criterios de inclusión: (1) amputados con edad igual o superior a 18 años, (2) amputación mayor de miembro inferior debido a la etiología vascular, (3) que vivían en casa, (4) que recibían asistencia de un cuidador familiar para las actividades de vida diaria (AVD) en casa y (5) con capacidad cognitiva para entender. Los criterios de exclusión fueron los siguientes: (1) negativa a firmar el término de consentimiento informado, (2) independencia en todas las AVD y (3) vivir en casas de reposo e instituciones.

Se utilizó muestreo por conveniencia y se reclutó un total de 40 pacientes con amputación de miembro inferior mayor disvascular para el estudio. Todos los participantes del estudio estaban viviendo en casa en el momento de la recolección de datos. La recolección de datos fue planificada para un período de un año, con un total de 13 meses de recolección de datos para contactar a toda la población hospitalizable con amputación mayor de miembro inferior debido a la EAP que estaba siendo seguida en la clínica. Nuevos pacientes sin consulta programada fueron identificados e incluidos en el estudio. De la población accesible, cinco amputados fallecieron, cinco se negaron a participar, siete no cumplieron con los criterios de inclusión y ocho faltaron a las consultas y no reprogramaron.

### Instrumentos de recolección de datos

El estudio utilizó dos instrumentos:

a) Cuestionario sobre variables sociodemográficas y clínicas: Este instrumento fue desarrollado para este estudio y consta de dos partes, una relacionada con las variables sociodemográficas: sexo, edad, nivel educativo, estado de empleo en el momento de la cirugía, domicilio y destino después del alta, y quién lo apoyó después del alta. En cuanto a las variables clínicas incluidas en el instrumento, antecedentes clínicos, fecha de la cirugía, nivel de la amputación, fecha del alta y amputación contralateral anterior.

*b) Self-Care Dependency Evaluation Form* (SCDEF) *Short Version* ^(^ 18 ^)^ : Este formulario determina las habilidades de autocuidado de pacientes con dependencias. El instrumento consta de 27 ítems que reportan actividades de evaluación de autocuidado asignadas a más de 10 dominios de autocuidado: caminar, transferirse, girar, levantarse, ir al baño, alimentarse, arreglarse, vestirse y desvestirse, bañarse y tomar medicamentos. Cada actividad de autocuidado es puntuada en una escala Likert de cuatro puntos: (1) dependiente, no participa, (2) necesita ayuda de otra persona, (3) necesita dispositivos de asistencia y (4) totalmente independiente, permitiendo una evaluación general de la dependencia de autocuidado por dominio y actividad. Este instrumento ha sido ampliamente utilizado en la evaluación de la discapacidad del autocuidado y ha demostrado tener buenas propiedades métricas ^(^ 18 ^)^ . 

### Consideraciones éticas

Las premisas deontológicas recomendadas por la ética aplicada a la investigación con seres humanos se siguieron durante el proceso metodológico; el estudio fue aprobado por el comité de ética del hospital donde se realizó la recolección de datos, de acuerdo con la Declaración de Helsinki. Cada formulario fue codificado con un número para garantizar la confidencialidad. Los participantes que accedieron a participar en el estudio firmaron un término de consentimiento informado.

### Análisis de datos

 Los análisis estadísticos se realizaron con el *software* IBM SPSS (versión 29). Las variables continuas se expresan como media ± desviación estándar, y las variables categóricas se expresan como porcentajes o número de observaciones. Se realizó un análisis de agrupamiento en dos etapas para identificar grupos distintos dentro de la muestra basados en los niveles de dependencia de autocuidado. El proceso de fusión se basó en el Criterio Bayesiano de Schwarz (CBS), que ayudó a determinar la estructura óptima de agrupamiento. La calidad y la validez de los agrupamientos se evaluaron mediante puntuaciones de silueta que varían de -1 a 1. Valores más altos de silueta indican agrupamientos más bien definidos. Además, se analizaron los valores de BIC para confirmar el número ideal de *clusters* . Los agrupamientos resultantes fueron interpretados mediante el análisis de las medias y distribuciones de las puntuaciones de dependencia de autocuidado en cada agrupamiento. 

## Resultados

### Características sociodemográficas y clínicas de los participantes

 De los 40 (100%) individuos con amputación mayor de miembros inferiores de origen vascular; la mayoría eran hombres (80%) y el 20% eran mujeres. La distribución por edades fue escalonada, siendo el grupo de edad más común entre 76 y 80 años (27,5%) y los grupos de edad menos comunes entre 46 y 50 años (2,5%) y 51 y 55 años (2,5%). En cuanto al nivel educativo, el 70% tenía cuatro años de educación primaria, mientras que solo el 2,5% tenía educación universitaria. Un total del 21% de los amputados estaban jubilados en el momento de la cirugía y el 5,0% estaban empleados. En términos de situación de vida, el 70% de los amputados vivía en una casa con otra persona y el 5% vivía solo. Después de la cirugía de amputación, el 85% de los individuos regresó a casa después del alta hospitalaria y el 24% recibió apoyo de un cónyuge o pareja después de regresar a casa con una amputación. La Tabla 1 muestra las características sociodemográficas de los participantes investigados. 

 La Tabla 2 muestra las variables clínicas de los 40 (100%) individuos amputados. Todos los individuos tenían EAP y el 57,5% también tenía DM asociada con EAP. En cuanto al número de comorbilidades, el 32,5% de los individuos presentó tres comorbilidades, el 32,5% dos y solo el 12,5% una comorbilidad (PAD). Un total de 77,5% de los amputados tuvo una amputación por encima de la rodilla, el 22,5% tuvo una amputación por debajo de la rodilla, el 7,5% tuvo una amputación anterior del dedo contralateral y el 7,5% tuvo una amputación transmetatarsiana anterior contralateral. Cabe señalar que el 15% de los participantes sufrió una amputación hace menos de un año, el 50% entre uno y cinco años y el 14% hace más de cinco años. En términos de dispositivos de asistencia usados para la movilidad, el 72,5% de los amputados usaba solo una silla de ruedas para la movilidad, el 10% tenía una prótesis pero seguía usando una silla de ruedas para la movilidad, el 7,5% tenía una prótesis pero necesitaba usar muletas, el 5% tenía una prótesis y otro 5% usaba una silla de ruedas y muletas para la movilidad. 

tabla 1

**Tabla 1 -:** Distribución numérica y porcentaje de participantes, según variables sociodemográficas (n = 40). Vila Nova de Gaia, VNG, Portugal, 2022-2023

Variables	Participantes (n)	Porcentaje (%)
**Género**		
Masculino	32	80
Feminino	8	20
**Edad (años)**		
46-50	1	2.5
51-55	1	2.5
56-60	3	7.5
61-65	6	15.0
66-70	9	22.5
71-75	4	10.0
75-80	11	27.5
> 81	5	12.5
**Nivel educativo**		
Sin educación formal	1	2.5
4 años (1ª etapa de la primaria)	28	70.0
6 años (2ª etapa de la primaria)	3	7.5
9 años (3ª etapa de la primaria)	3	7.5
12 años (secundaria)	4	10.0
Graduado	1	2.5
**Estado de empleo en el momento de la cirugía**		
Empleado activo	2	5.0
Desempleado	1	2.5
Jubilado	21	52.5
Jubilación anticipada	16	40.0
**Cómo vive en casa**		
Solo	2	5.0
Vive con una persona	28	70.0
Vive con dos o más personas	10	25.0
**Destino después del alta**		
Casa	34	85.0
Casa de la hermana	1	2.5
Rehabilitación con internación de corto plazo	4	10.0
Casa de la hija	1	2.5
**Quién da soporte después del alta**		
Amigo/vecino	1	2.5
Hijos	11	27.5
Cónyuge/compañero(a)	24	60.0
Hermano/Hermana	4	10.0

* *n = Número de participantes

tabla 2

**Tabla 2 -:** Distribución numérica y porcentaje de participantes, según las variables clínicas (n = 40). Vila Nova de Gaia, VNG, Portugal, 2022-2023

Variables	Participantes (n)	Porcentaje (%)
**Historial clínico**		
Enfermedad arterial periférica	40	100.0
Dislipidemia	2	5.0
Hipertensión	20	50.0
Tabaquista	7	17.5
Enfermedad pulmonar obstructiva crónica	7	17.5
Enfermedad cardíaca	7	17.5
Insuficiencia renal	7	17.5
Diabetes	18	45.0
Retinopatía diabética	1	2.5
Anemia crónica	1	2.5
Insuficiencia respiratoria	1	2.5
Vejiga hiperactiva	1	2.5
Artritis reumatoide	1	2.5
**Total de comorbilidades**		
Una comorbilidad	5	12.5
Dos comorbilidades	13	32.5
Tres comorbilidades	13	32.5
Cuatro comorbilidades	6	15.0
Cinco comorbilidades	3	7.5
**Nivel de amputación**		
Amputación transfemoral	31	77.5
Amputación transtibial	9	22.5
**Amputación contralateral anterior**		
Ausente	34	85.0
Amputación de dedo	3	7.5
Amputación transmetatarsiana	3	7.5
**¿Cuánto tiempo ha pasado desde la amputación?**		
< 1 año	6	15.0
1-5 años	20	50.0
> 5 años	14	35.0
**Dispositivos de asistencia para la movilidad**		
Silla de ruedas (solamente)	29	72.5
Silla de ruedas y muletas	2	5.0
Prótesis y silla de ruedas	4	10.0
Prótesis y muletas	3	7.5
Prótesis (solamente)	2	5.0

* *n = Número de participantes

### Grado de dependencia por dominio de autocuidado

La evaluación de la dependencia en el autocuidado de la AMI se realizó de acuerdo con los 10 dominios incluidos en la versión corta del SCDEF, en cuanto al dominio de autocuidado “caminar”, “transferirse”, “girar”, “levantarse”, “usar el baño”, “alimentarse”, “arreglarse”, “vestirse y desvestirse”, “bañarse” y “tomar medicamentos”. El autocuidado se clasifica mediante una escala Likert de cuatro puntos en cada dominio de autocuidado: (1) dependiente no participa, (2) necesita ayuda de otra persona, (3) necesita dispositivos de asistencia y (4) totalmente independiente, lo que ayuda a evaluar el nivel de dependencia y las necesidades del amputado MI en relación con las actividades de la vida diaria.

En el autocuidado “caminar”, tres actividades fueron incluidas en este dominio para evaluar el autocuidado, es decir, mantener el cuerpo en posición erguida, de pie sin asistencia alguna de dispositivos de asistencia u otra persona, utilizando dispositivos de asistencia que incluyen muletas o prótesis. Respecto a los tres dominios incluidos, el 5% de los amputados IM son capaces de mantenerse en pie sin ningún tipo de ayuda de dispositivos de asistencia u otra persona, el 70% necesita utilizar dispositivos de asistencia que incluyen muletas o prótesis y el 12,5% no puede mantenerse de pie. respectivamente.

En cuanto a la actividad de subir y bajar escaleras, el 70% de los amputados MI son incapaces de hacerlo, el 22,5% suben y bajan escaleras usando muletas y prótesis y el 7,5% pueden subir y bajar escaleras con la ayuda de una persona. En la actividad de caminar distancias medias, el 72,5% de los amputados MI logran hacerlo con la ayuda de muletas, prótesis o silla de ruedas; el 7,5% de los participantes del estudio no pudieron caminar distancias, incluso con ayuda o dispositivos de asistencia.

En el autocuidado “transferencia”, que evalúa la capacidad de los amputados MI de transferirse de la cama a la silla/sillón y de la silla/sillón a la cama, el 62,5% requiere el uso de dispositivos de asistencia para realizar la transferencia, el 27,5% no pueden realizar la transferencia de manera independiente o con el uso de dispositivos de asistencia y requieren la ayuda de otra persona para realizar la transferencia. Solo el 10% de los amputados MI lograron transferirse de la cama a la silla/sillón y de la silla al sillón de forma independiente, sin el uso de dispositivos de asistencia o ayuda de otra persona.

En cuanto al dominio de autocuidado “girar”, que evalúa si la persona con amputación de miembro inferior mueve el cuerpo de un lado a otro, los resultados muestran que el 65% de los amputados necesitan dispositivos de asistencia, el 7,5% logran girarse solo con la ayuda de otra persona y el 27,5% son totalmente independientes en este dominio. En relación con el autocuidado “levantarse”, que se refiere a la capacidad de levantar una parte del cuerpo, la mayoría de los participantes, el 52,5% de los amputados, es independiente, el 40,0% necesitan dispositivos de asistencia y el 7,5% necesitan la ayuda de otra persona.

En el autocuidado “uso del baño”, en relación con la actividad de posicionarse en el inodoro o en la silla higiénica, el 77,5% de los amputados MI necesita dispositivos de asistencia; en la habilidad de levantarse del inodoro, el 72,5% de los participantes necesita dispositivos de asistencia; en relación con la actividad de arreglar la ropa después de la higiene personal, el 27,5% necesita dispositivos de asistencia y el 45% son completamente independientes.

En cuanto al autocuidado “alimentarse”, la mayoría de los amputados MI, el 92,5%, son independientes para abrir recipientes, el 97,5% son independientes para sostener un vaso o una taza, pero el 87,5% necesita ayuda de otra persona para preparar alimentos para comer.

El cuidado de las uñas es la actividad relacionada con el autocuidado “arreglarse” con mayor dependencia, con el 97,5% necesitando la ayuda de otra persona; en cuanto a las actividades de peinarse, aplicar desodorante y mantener la higiene bucal, la mayoría de los participantes es independiente.

El autocuidado “vestirse y desvestirse” incluye cinco actividades de autocuidado, el 67,5% de los amputados MI necesita la ayuda de otra persona para atarse los zapatos y el 65% necesita ayuda para ponerse los calcetines. Un total del 82,5% eran totalmente independientes para vestirse, el 52,5% necesitaba la ayuda de otra persona para elegir la ropa y el 47,5% necesitaba la ayuda de otra persona para vestir la parte inferior del cuerpo.

Una de las actividades de autocuidado con mayor nivel de dependencia en la persona con AMI es la actividad de autocuidado “bañarse”, relacionada con la obtención de artículos para el baño, el 92,5% de los amputados MI en el estudio necesitan la ayuda de otra persona, en la capacidad de lavar el cuerpo, un total del 85,0% necesita la ayuda de otra persona. Un total del 55% de los amputados MI eran completamente independientes en abrir la llave para el baño. Con respecto a la actividad de autocuidado “tomar medicación”, el 85,0% de los amputados MI necesitan la ayuda de otra persona para preparar la medicación, y con respecto a la actividad “tomar medicación”, el 82,5% son independientes.

tabla 3

**Tabla 3 -:** Media, desviación estándar, mínimo y máximo para cada dominio del autocuidado. Vila Nova de Gaia, VNG, Portugal, 2022-2023

Dominios del autocuidado	n*	Mínimo	Máximo	Media	Desviación Estándar
Caminar	40	1.00	3.33	2.28	0.59
Transferirse	40	2.00	4.00	2.83	0.59
Girar	40	2.00	4.00	3.20	0.56
Levantarse	40	2.00	4.00	3.45	0.64
Usar el baño	40	1.00	3.33	2.83	0.65
Alimentarse	40	1.75	4.00	3.45	0.32
Arreglarse	40	1.25	3.50	3.37	0.48
Vestirse y desvestirse	40	1.20	3.80	2.81	0.61
Bañarse	40	1.00	3.33	2.41	0.47
Tomar medicamentos	40	1.50	4.00	3.19	0.75
Nivel general de dependencia	40	1.38	3.52	2.99	0.43

* n = Número de participantes del estudio

 Para crear una variable general, los ítems de cada dominio se combinaron. La Tabla 3 muestra la conversión de las diez variables calculadas asociadas a diferentes actividades de autocuidado en una única variable compuesta llamada nivel de dependencia general. 

 Según los resultados presentados en la Tabla 4 , el dominio de autocuidado con un alto nivel de dependencia es el autocuidado “caminar” con una media de 2,28 y una desviación estándar de 0,59, seguido del autocuidado “bañarse” con una media de 2,41 y una desviación estándar de 0,47; autocuidado “vestirse y desvestirse” con una media de 2,81 y una desviación estándar de 0,61%; autocuidado “usar el baño” con una media de 2,83 y una desviación estándar de 0,65 y autocuidado “transferirse” con una media de 2,83 y una desviación estándar de 0,56%. 

Considerando los dominios de autocuidado con menores niveles de dependencia, el autocuidado “alimentarse” presenta el menor nivel de dependencia con una media de 3,45% y una desviación estándar de 0,32%, seguido por el autocuidado “levantarse” con una media de 3,45% y una desviación estándar de 0,64%; autocuidado ” vestirse y desvestirse” con una media de 3,37% y una desviación estándar de 0,48%; autocuidado “girar” con una media de 3,20% y una desviación estándar de 0,56%; y autocuidado “tomar medicamentos” con una media de 3,19% y una desviación estándar de 0,75%.

El nivel global de dependencia de autocuidado de los amputados de MI en este estudio presentó una media de 2,99% con una desviación estándar de 0,43%, y casi todos los participantes necesitaron asistencia en los dominios de autocuidado evaluados. Los tipos de dispositivos de asistencia usados por los amputados MI para auxiliar en las actividades de autocuidado incluían dispositivos ambulatorios, como sillas de ruedas, muletas y prótesis. Los amputados MI usaban bancos de ducha, barras de apoyo y sillas de ducha como dispositivos de asistencia para ayudar en el baño. En el dormitorio, utilizaban el cabecero y los bordes de la cama para ayudar en el giro, levantamiento y transferencia, y no usaban ningún dispositivo de asistencia para adaptarse a la cama y ayudar en las actividades de autocuidado.

 El análisis de *cluster* en dos etapas identificó dos *clusters* distintos dentro de la muestra de individuos con amputación disvascular de miembro inferior mayor, con altas puntuaciones de silueta indicando buena cohesión y separación entre los *clusters* . El *cluster* 1 incluyó una proporción mayor de pacientes más mayores, frágiles y dependientes. La Tabla 4 proporciona una descripción detallada de los *clusters* formados, incluyendo sus características sociodemográficas y clínicas, así como su nivel de dependencia en el autocuidado. 

tabla 4

**Tabla 4:** - Características del cluster. Vila Nova de Gaia, VNG, Portugal, 2022-2023

	Cluster 1 (n*=9)	Cluster 2 (n*=31)
Masculino	88.8% (8)	77.4% (24)
Escuela primaria	100% (9)	61.3% (19
Amputación transfemoral	88.8% (8)	74.2% (23)
Andar	M=1.44	M=2.53
Transferirse	M=2.00	M=3.06
Uso del baño	M=1.85	M=3.11
Alimentarse	M=3.22	M=3.52
Arreglarse	M=2.92	M=3.50
Vestirse y devsetirse	M=2.09	M=3.01
Levantarse	M=2.78	M=3.65
Girar	M=2.67	M=3.35

 Mayor autonomía en el uso del baño, deambulación y transferencia de la cama a la silla se mostraron como actividades de autocuidado con mejor capacidad para predecir la autonomía de este tipo de pacientes ( Figura 1 ). 

## Discusión

Hasta donde sabemos, este estudio es el primero en examinar la dependencia de autocuidado en actividades de la vida diaria en individuos que han sufrido una amputación de miembro inferior mayor de origen vascular. Este estudio utilizó un instrumento que permite una evaluación priorizada y operacional de las competencias de autocuidado en individuos con amputaciones mayores de miembros inferiores, divididas en 10 dominios.

 Al centrarse en individuos que han sufrido una amputación mayor y disvascular del miembro inferior, este estudio proporciona información valiosa sobre sus características sociodemográficas y clínicas. También examina hasta qué punto dependen de asistencia para las actividades de la vida diaria. En total, el 80% de la muestra de 40 personas eran hombres y el 20% eran mujeres. Estudios anteriores han reportado que los hombres tienen un riesgo mayor de amputación de miembros inferiores que las mujeres ^(^ 19 ^)^ , lo que puede explicarse por tasas más altas de enfermedad arterial periférica, neuropatía periférica y tabaquismo en hombres que en mujeres ^(^ 20 ^-^ 21 ^)^ . La menor incidencia de amputación de miembros inferiores en mujeres con enfermedad vascular puede explicarse por el efecto del estrógeno en la reducción de la patología vascular ^(^ 22 ^)^ . 

**Figura 1:** - Predictores de cluster

 En cuanto a la edad de los amputados de miembros inferiores incluidos en nuestro estudio, el grupo de edad más común fue entre 76 y 80 años, representando el 27,5% de la muestra del estudio, y el grupo de edad menos común fue entre 46 y 50 años, representando solo el 2,5% de los participantes. Evidencias anteriores sugieren que la mayoría de las amputaciones actuales son secundarias a enfermedades disvasculares, y la prevalencia de EAP aumenta significativamente con la edad, tanto en hombres como en mujeres. En personas menores de 50 años, la prevalencia es del 15% al 20%, aumentando del 15% al 20% a los 80 años de edad ^(^ 23 ^-^ 24 ^)^ . 

 En el presente estudio, el 40% de los amputados de miembros inferiores con disfunción vascular estaban jubilados precozmente antes de la amputación. Estudios anteriores han reportado que la claudicación intermitente es una manifestación de enfermedad arterial periférica, resultando en movilidad y calidad de vida reducidas ^(^ 25 ^)^ . Incluso en pacientes con enfermedad arterial periférica con o sin síntomas atípicos, el deterioro funcional está presente; la fragilidad es común en pacientes con EAP sintomática y está asociada con el deterioro de la marcha ^(^ 25 ^-^ 26 ^)^ . 

 De acuerdo con hallazgos anteriores, los pacientes mayores tienen menos probabilidad de movilizarse con prótesis que sus colegas más jóvenes, ya que el nivel de amputación de los pacientes con amputación transtibial es dos veces más probable de movilizarse con prótesis que los pacientes con amputación transfemoral ^(^ 27 ^)^ . En la muestra de nuestro estudio, el 72,5% de los amputados de miembros inferiores tenían más de 65 años de edad, el 77,5% tenían una amputación transfemoral y el 72,5% estaban confinados a una silla de ruedas. Investigadores ^(^ 28 ^)^ han reportado que, en un período de 3 meses después de la amputación, todos los grupos de edad de amputados de miembros inferiores presentaron las puntuaciones más bajas de funciones físicas y, 12 meses después de la amputación, hay una diferencia entre los grupos de edad en un nivel funcional, con una pérdida obvia de función en los pacientes más viejos. Estos pacientes necesitan un enfoque especial que requiere rehabilitación diaria para recuperar sus funciones físicas básicas. La función física en amputados de miembros inferiores también puede verse afectada por el número de comorbilidades presentes. 

 Nuestros resultados mostraron que el 65% de los amputados de miembros inferiores en nuestro estudio tenían al menos dos o tres comorbilidades. Los adultos que han sufrido amputación de miembros inferiores debido a enfermedades vasculares generalmente tienen múltiples problemas de salud ^(^ 29 ^)^ . La presencia de múltiples comorbilidades en pacientes con enfermedad arterial periférica avanzada generalmente resulta en niveles elevados de fragilidad ^(^ 30 ^)^ . 

 Resultados físicos pobres, incluyendo problemas de equilibrio, disminución de la fuerza y movilidad limitada o capacidad para caminar, son comúnmente observados en pacientes con amputaciones disvasculares de miembros inferiores. Las personas con amputaciones disvasculares de miembros inferiores a menudo presentan una serie de problemas de salud que afectan su bienestar físico y su capacidad para realizar tareas diarias ^(^ 10 ^,^ 31 ^)^ . Nuestro estudio encontró que, estadísticamente, los dominios de autocuidado con mayor dependencia en amputados de miembros inferiores mayores con disfunción vascular fueron “caminar”, “bañarse”, “vestirse y desvestirse”, “usar el baño”, “transferirse”, siendo el autocuidado de caminar el de mayor dependencia en nuestros participantes. Caminar y subir escaleras son las actividades más difíciles para individuos que han sufrido amputación de miembros inferiores. Los individuos con amputaciones transfemorales son particularmente afectados y no logran recuperar la capacidad de realizar actividades básicas e instrumentales de la vida diaria ^(^ 32 ^)^ . 

 En cuanto a las áreas de autocuidado con mayor dependencia de AVD identificadas en nuestro estudio, la literatura indica que los pacientes con más de 12 meses después de la amputación necesitan más asistencia con higiene personal, baño, vestirse e ir al baño ^(^ 28 ^)^ . El declive más significativo en la movilidad, particularmente en la marcha independiente, fue observado 12 meses después de la amputación. La capacidad del paciente para caminar sin ayuda depende de la presencia de un miembro protésico ^(^ 28 ^)^ . De todos los participantes de nuestro estudio, solamente el 5% logró caminar usando solo prótesis. Doce meses después de la amputación, la mayoría de los amputados de miembros inferiores aún no logra recuperar el mismo nivel de independencia en sus actividades diarias ^(^ 28 ^)^ . 

 Los enfermeros juegan un papel importante en alentar a los amputados de miembros inferiores con disfunción vascular para que recuperen la independencia en las actividades de autocuidado. Según nuestro análisis de agrupamiento, una mayor autonomía de autocuidado en el uso del baño, en caminar y en la transferencia de la cama a la silla puede predecir la autonomía en este tipo de pacientes. Estudios anteriores mostraron que caminar de manera independiente ayuda a recuperar la independencia en la realización de habilidades básicas y actividades de la vida diaria ^(^ 33 ^)^ . Las intervenciones de rehabilitación domiciliaria para amputados de miembros inferiores con disfunción vascular con ejercicios para mejorar el equilibrio en pie, la capacidad de sentarse y levantarse, las transferencias y la movilidad ayudan a estos amputados a recuperar la movilidad y la independencia funcional más pronto, contribuyendo a mejorar la movilidad y la calidad de vida ^(^ 34 ^)^ . 

Este estudio tiene algunas limitaciones, como el pequeño tamaño de la muestra de pacientes no probabilísticos de un solo hospital en el norte de Portugal. Por lo tanto, se debe tener cautela al generalizar los resultados. Para mejorar estudios futuros, se recomienda aumentar el tamaño de la muestra y extender la duración del estudio. A pesar de ciertas limitaciones, este estudio arroja luz sobre el impacto de la amputación de miembros inferiores con disfunción vascular grave en las actividades de la vida diaria. Estos hallazgos pueden proporcionar consideraciones para la planificación de futuras investigaciones sobre la dependencia de una persona con amputación de miembro inferior mayor disvascular en actividades de la vida diaria y para el desarrollo de intervenciones y programas para ayudar a capacitar a pacientes, cuidadores y familiares.

## Conclusión

Este estudio demostró que el dominio del autocuidado con mayor nivel de dependencia es “caminar” y el más bajo es el “alimentarse”. Una mayor autonomía en el uso del baño, caminar y pasar de la cama a la silla resultaron ser actividades de autocuidado con mayor capacidad para predecir la autonomía del paciente. Os pacientes con este tipo de amputación tiene más de 65 años de edad; solo el 5% es capaz de deambular con prótesis y tiene algún nivel de dependencia en los dominios de autocuidado relacionados con las actividades de la vida diaria.

Con el uso de una herramienta de evaluación confiable para evaluar el nivel de dependencia en los diferentes dominios de autocuidado de una persona con amputación de miembro inferior mayor de origen vascular, es importante identificar sus necesidades en relación con las actividades de autocuidado. Esto puede ayudar a entender el impacto funcional y la discapacidad que una amputación disvascular trae y el nivel de asistencia que la persona con este tipo de amputación necesita para sus actividades diarias.

Actualmente, hay una tendencia de alta precoz con retorno anticipado a la comunidad o al hogar cuando el amputado aún es totalmente dependiente de otras personas. Evaluar la dependencia de una persona con una amputación de miembro inferior mayor de origen vascular en las actividades de la vida diaria es esencial para diseñar intervenciones y programas educativos que mejoren su capacidad para realizar actividades básicas de la vida diaria. Las intervenciones de rehabilitación deben continuar después del alta con ejercicios para mejorar la capacidad funcional y la independencia en las actividades de autocuidado. Los cuidadores familiares deben estar involucrados desde el inicio de estas intervenciones y programas para ayudar y apoyar al amputado de miembro inferior a recuperar algún nivel de independencia en la realización de actividades de la vida diaria, contribuyendo así a una mejor calidad de vida.

# Translation: Avaliação da dependência do autocuidado em pessoas com amputações dos membros inferiores: um estudo exploratório

**DOI:** 10.1590/1518-8345.7424.4333

**Published:** 2024-10-02

**Authors:** Diana Fonseca Rodrigues, Paulo Alexandre Puga Machado, Teresa Martins, António Luís Rodrigues Faria de Carvalho, Cristina Maria Correia Barroso Pinto

**Affiliations:** ^1^ Center for Health Technology and Services Research, Porto, Portugal.; ^2^ University of Porto, School of Medicine and Biomedical Sciences, Porto, Portugal.; ^3^ Nursing School of Porto, Porto, Portugal.; ^4^ Bolsista da Foundation for Science and Technology (FCT), Portugal.

**Keywords:** Atividades Diárias, Amputação, Pessoa com Deficiência, Membro Inferior, Autocuidado, Doença Vascular

## Abstract

identificar as características sociodemográficas e clínicas de pessoas com amputação maior de membro inferior, de origem vascular, e avaliar o nível de dependência e autonomia nas atividades de autocuidado, no domicilio.

estudo quantitativo, exploratório, transversal e descritivo. A amostra por conveniência foi constituída por 40 participantes. Para a coleta de dados utilizou-se um questionário sociodemográfico e a versão curta do Formulário de Avaliação de Dependência de Autocuidado.

dos 40 (100%) participantes a maioria era do sexo masculino; 75% tinham idade superior a 65 anos de idade e 77,5% foram submetidos a amputação transfemoral. Em relação ao nível de dependência predominavam as atividades relacionadas ao autocuidado: “andar”, “tomar banho”, “vestir-se e despir-se”, “usar o banheiro” e “transferir-se” e quanto a autonomia, 72,5% estavam confinados a uma cadeira de rodas.

o domínio de autocuidado com maior nível de dependência é *“andar”,* e o menor *, “alimentar-se”.* Maior autonomia no uso do banheiro, deambulação e transferência da cama para a cadeira mostraram-se atividades de autocuidado com melhor capacidade de predizer a autonomia do paciente.

## Destaques

**(1)** A amputação maior de membro inferior de causa vascular interfere nas atividades da vida diária.

**(2)** Autocuidado com maior nível de dependência é *“andar”* .

**(3)** Autocuidado menor nível de dependência é *“alimentar-se”* .

**(4)** Desenvolver futuras intervenções sobre o nível de dependência de indivíduos com amputação de causa vascular.

## Introdução

 A amputação de membro inferior (AMI) altera e afeta vários aspectos da vida da pessoa amputada e apresenta desafios para ela, mostrando, sobretudo, um impacto global e significativo na morbidade dos amputados ^(^ 1 ^)^ . A causa mais comum de amputação de membro inferior (AMI) é a amputação de causa vascular, definida como secundária às complicações de doença arterial periférica, diabetes mellitus (DM) ou ambas, com mais de 80% das amputações de membro inferior devido à etiologia vascular ^(^ 2 ^-^ 3 ^)^ . A doença arterial periférica (DAP) ocorre em pessoas com DM. Ela geralmente é assintomática, com prevalência estimada de 10% a 20%. Além disso, a presença do DM leva à inflamação crônica e ao estresse oxidativo, que exacerbam ainda mais a DAP e retardam a capacidade do corpo de reparar o tecido isquêmico. A presença desses processos patológicos pode levar a uma maior incidência de complicações, incluindo dor, redução da funcionalidade e aumento do risco de morte ^(^ 4 ^)^ . 

 Uma amputação de membro inferior é um evento que muda a vida e pode ter repercussão negativa na saúde física e mental de uma pessoa ^(^ 2 ^)^ . Apenas dois terços dos pacientes são encaminhados a centros de adaptação de membros após a amputação, sendo que somente 40% desses pacientes acabam recebendo um membro protético. Como resultado, a maioria dos pacientes permanece dependente de cadeira de rodas após a amputação ^(^ 5 ^-^ 6 ^)^ . Estudos observacionais mostram que a capacidade funcional, 12 meses após a AMI de causa vascular, compromete a função física e que apenas 39% dos pacientes retornam aos níveis anteriores de mobilidade ^(^ 7 ^)^ . Os resultados da reabilitação após a amputação de causa vascular são ruins, com os pacientes apresentando maior incapacidade do que 95% da população em geral ^(^ 8 ^)^ . 

 Pessoas com DAP e DM que sofrem amputação geralmente têm problemas de saúde pré-existentes e podem apresentar comprometimento cognitivo ^(^ 9 ^)^ . A perda de membros de causa vascular está associada a alta prevalência de múltiplos problemas de saúde que podem afetar negativamente o bem-estar geral e a capacidade funcional de um indivíduo ^(^ 10 ^)^ . As amputações de membros inferiores (AMIs) de causa vascular submetidos à amputação transfemoral têm um risco maior de mortalidade no primeiro ano após a cirurgia, indicando a presença de doença vascular mais grave. A fragilidade dessa população se reflete em uma taxa de mortalidade mais alta na maioria dos pacientes idosos ^(^ 11 ^)^ . 

 A mortalidade é particularmente alta em pacientes submetidos à amputação transfemoral no primeiro ano após a cirurgia. As taxas de mortalidade mais altas observadas em idosos, especialmente entre aqueles com doença cardíaca grave e submetidos à hemodiálise, indicam a vulnerabilidade dessa população. A amputação de causa vascular do membro inferior surgiu como um dos principais contribuintes para a prevalência global de incapacidade ^(^ 11 ^-^ 12 ^)^ . Pessoas com amputações de membros inferiores de causa vascular enfrentam desafios para participar de atividades físicas devido a doenças crônicas, deficiência grave e fatores psicológicos e sociais não abordados ^(^ 13 ^)^ . Amputados de membros inferiores (MI) com condições vasculares relatam um declínio em suas habilidades funcionais, levando a uma percepção de capacidade física e independência reduzidas ^(^ 14 ^)^ . 

 A AMI é responsável por deficiências físicas que podem limitar a capacidade de um amputado de desenvolver as atividades na vida diária. É possível que os pacientes sofram uma perda debilitante de independência, ocasionando, eventualmente, mudanças físicas, comportamentais e psicológicas. A mobilidade é alterada em todos os amputados, a atividade física é reduzida e é difícil para os pacientes permanecerem ativos devido ao aumento do gasto de energia ^(^ 1 ^)^ . Os amputados de membros inferiores enfrentam um problema funcional fundamental, além da limitação do movimento independente na vida diária, levando ao aumento da dependência ^(^ 1 ^)^ . 

 Uma pessoa dependente é definida como aquela que tem a capacidade limitada ou a incapacidade de iniciar e desenvolver atividades importantes para o próprio bem-estar, para a saúde e a manutenção da vida sem a assistência de outra pessoa ^(^ 15 ^)^ . Isso inclui atividades da vida diária, como tomar banho, higiene pessoal, transferir-se, ir ao banheiro, caminhar, comer e se posicionar ^(^ 16 ^)^ . Para entender as verdadeiras necessidades de uma pessoa, não basta dizer que ela é dependente de outras pessoas para o autocuidado. Também o tipo, a quantidade e a natureza do apoio necessário podem variar conforme a área de dependência da pessoa e das habilidades do cuidador ^(^ 17 ^)^ . O uso de uma ferramenta de avaliação robusta e confiável que possa avaliar o nível de dependência de acordo com as diferentes áreas de autocuidado é muito importante para que os profissionais de saúde possam elaborar um plano de alta que seja centrado no paciente ^(^ 17 ^)^ . 

Este estudo faz parte de um projeto de pesquisa de doutorado mais amplo que identifica e reconhece o perfil da pessoa com amputação maior de membro inferior de causa vascular. Os objetivos deste estudo foram: identificar as características sociodemográficas e clínicas de pessoas com amputação de membro inferior maior, de origem vascular, e avaliar o nível de dependência e autonomia nas atividades de autocuidado, no domicílio.

Este estudo pode contribuir para o desenvolvimento de intervenções e programas para a capacitação de pessoas amputadas e cuidadores em relação à dependência do autocuidado relacionado à deficiência de uma amputação maior de membro inferior por doença arterial periférica na transição para o domicílio.

## Método

### Tipo e local do estudo

 Estudo quantitativo, exploratório, transversal e descritivo, conduzido de acordo com as diretrizes do *Strengthening the Reporting of Observational Studies in Epidemiology* (STROBE) para estudos observacionais, com o objetivo de avaliar a dependência do autocuidado nas atividades de vida diária da pessoa com amputação maior de membro inferior de origem vascular. A pesquisa faz parte de um estudo maior, exploratório, transversal e de métodos mistos, com um paradigma predominantemente qualitativo, como parte de um projeto de doutorado em enfermagem. O estudo foi realizado em uma consulta de hemodinâmica para acompanhamento de doenças vasculares no serviço de cirurgia vascular de uma unidade hospitalar do norte de Portugal. A coleta de dados ocorreu entre maio de 2022 e junho de 2023. 

### População e amostra

A população da amostra deste estudo foi selecionada entre os pacientes que compareceram à consulta de hemodinâmica para doença vascular em um serviço de cirurgia vascular em um hospital no norte de Portugal, onde o estudo foi realizado. Os pacientes com amputação maior de membro inferior de causa vascular e que compareceram à consulta foram recrutados para o estudo de acordo com os seguintes critérios de inclusão: (1) amputados com idade igual ou superior a 18 anos, (2) amputação maior de membro inferior devido à etiologia vascular, (3) que moravam em casa, (4) que recebiam assistência de um cuidador familiar para as atividades de vida diária (AVDs) em casa e (5) com capacidade cognitiva para entender. Os critérios de exclusão foram os seguintes: (1) recusa em assinar o termo de consentimento livre e esclarecido, (2) independência em todas as AVDs e (3) morar em casas de repouso e instituições.

A amostra por conveniência foi constituída por 40 pacientes com amputação maior de membro inferior maior de causa vascular recrutados para o estudo. Todos os participantes do estudo estavam morando em casa no momento da coleta de dados. A coleta de dados foi planejada para um período de um ano, com um total de 13 meses de coleta de dados, para entrar em contato com toda a população hospitalizada com amputação maior de membro inferior devido à DAP que estava sendo acompanhada na clínica. Novos pacientes sem consulta marcada foram identificados e incluídos no estudo. Da população acessível, cinco amputados morreram, cinco se recusaram a participar, sete não atenderam aos critérios de inclusão e oito faltaram às consultas e não remarcaram.

### Instrumentos de coleta de dados

O estudo utilizou dois instrumentos:

a) Questionário sobre variáveis sociodemográficas e clínicas: o instrumento foi desenvolvido para este estudo e consiste em duas partes, uma relacionada às variáveis sociodemográficas tais como sexo, idade, nível de escolaridade, status de emprego no momento da cirurgia, domicílio, destino após a alta, e quem o apoiou após a alta). As variáveis clínicas incluídas no instrumento foram: antecedentes clínicos, data da cirurgia, nível da amputação, data da alta e amputação contralateral anterior.
b) Self-Care Dependency Evaluation Form (SCDEF) Short Version ^(^ 18 ^)^ : Esse instrumento permite determinar as habilidades de autocuidado de pacientes com dependências. O instrumento contém 27 itens que relatam atividades de avaliação de autocuidado atribuídas a 10 domínios de autocuidado: andar, transferir-se, virar-se, levantar, ir ao banheiro, alimentar-se, arrumar-se, vestir-se e despir-se, tomar banho e tomar medicamentos. Cada atividade de autocuidado é pontuada em uma escala Likert de quatro pontos: (1) dependente, não participa, (2) precisa de ajuda de outra pessoa, (3) precisa de dispositivos de assistência e (4) totalmente independente, permitindo uma avaliação geral da dependência de autocuidado por domínio e atividade. Tal instrumento tem sido amplamente utilizado na avaliação da deficiência do autocuidado e apresenta boas propriedades métricas ^(^ 18 ^)^ .

### Aspectos éticos

As premissas deontológicas recomendadas pela ética aplicada à pesquisa com seres humanos foram seguidas durante o processo metodológico; o estudo foi aprovado pelo comitê de ética do hospital onde se realizou a coleta de dados, de acordo com a Declaração de Helsinki. Cada formulário foi codificado com um número para garantir a confidencialidade. Os participantes que concordaram em participar do estudo assinaram o Termo de Consentimento Livre e Esclarecido.

### Análise de dados

 As análises estatísticas foram realizadas com o software IBM SPSS (versão 29). As variáveis contínuas são expressas como média ± desvio-padrão, e as variáveis categóricas são expressas como porcentagens ou número de observações. Elaborou-se uma análise de agrupamento em duas etapas para identificar grupos distintos dentro da amostra com base nos níveis de dependência de autocuidado. O processo de fusão foi baseado no Critério Bayesiano de Schwarz (CBS), que ajudou a determinar a estrutura ideal de agrupamento. A qualidade e a validade dos agrupamentos foram avaliadas por meio de pontuações de silhueta que variam de -1 a 1. Valores mais altos de silhueta indicam agrupamentos mais bem definidos. Além disso, os valores de BIC foram analisados para confirmar o número ideal de *clusters* . Os agrupamentos resultantes foram interpretados por meio da análise das médias e distribuições das pontuações de dependência de autocuidado em cada agrupamento. 

## Resultados

### Características sociodemográficas e clínicas dos participantes

 Dos 40 (100%) indivíduos com amputação maior de membros inferiores de origem vascular, a maioria eram homens (80%) e 20%, mulheres. A distribuição etária foi intervalada, sendo a faixa etária mais frequente entre 76 e 80 anos (27,5%) e a menos, entre 46 e 50 anos (2,5%) e 51 e 50 anos (2,5%). Em relação ao nível de escolaridade, 70% tinham quatro anos de educação básica, enquanto apenas 2,5% tinham educação universitária. Um total de 21% dos amputados estavam aposentados no dia da cirurgia e 5%, empregados. Em termos de situação de vida, 70% dos amputados viviam em uma casa com outra pessoa e 5%, sozinhos. Após a cirurgia de amputação, 85% dos indivíduos voltaram para casa após a alta hospitalar e 24% receberam apoio de um cônjuge ou parceiro após voltarem para casa com uma amputação. A Tabela 1 mostra as características sociodemográficas dos participantes investigados. 

 A Tabela 2 mostra as variáveis clínicas dos 40 (100%) indivíduos amputados. A totalidade dos indivíduos tinham DAP e 57,5% também tinham DM associada à DAP. Com relação ao número de comorbidades, 32,5% dos indivíduos tinham três comorbidades, 32,5%, duas, e apenas 12,5%, uma comorbidade (DAP). Um total de 77,5% dos amputados tinham uma amputação acima do joelho, 22,5%, abaixo do joelho, 7,5% uma amputação contralateral anterior do dedo e 7,5%, uma amputação transmetatarsal contralateral anterior. Cabe ressaltar que 15% dos participantes sofreram a amputação há menos de um ano, 50%, de um a cinco anos e 14%, por mais de cinco anos. Em termos de dispositivos de assistência usados para mobilidade, 72,5% dos amputados usavam apenas uma cadeira de rodas para mobilidade, 10% tinham uma prótese, mas continuavam a usar uma cadeira de rodas para mobilidade, 7,5% tinham uma prótese, mas precisavam usar muletas, 5% tinham uma prótese e outros 5% usavam cadeira de rodas e muletas para mobilidade. 

**Tabela 1 -:** Distribuição numérica e porcentual dos participantes, segundo as variáveis sociodemográficas (n = 40). Vila Nova de Gaia, VNG, Portugal, 2022-2023

Variáveis	Participantes (n)	Porcentagem (%)
**Sexo**		
Masculino	32	80
Feminino	8	20
**Idade (anos)**		
46-50	1	2.5
51-55	1	2.5
56-60	3	7.5
61-65	6	15.0
66-70	9	22.5
71-75	4	10.0
75-80	11	27.5
> 81	5	12.5
**Nível educacional**		
Sem educação formal	1	2.5
4 anos (1ª etapa da educação básica)	28	70.0
6 anos (2ª etapa da educação básica)	3	7.5
9 anos (3ª etapa da educação básica)	3	7.5
12 anos (ensino médio)	4	10.0
Graduado	1	2.5
**Status de emprego no momento da cirurgia**		
Funcionário ativo	2	5.0
Desempregado	1	2.5
Aposentado	21	52.5
Aposentado precoce	16	40.0
**Como vive em casa**		
Sozinho	2	5.0
Vive com uma pessoa	28	70.0
Vive com duas ou mais pessoas	10	25.0
**Destino após a alta**		
Própria casa	34	85.0
Casa da irmã	1	2.5
Reabilitação com internação de curto prazo	4	10.0
Casa da filha	1	2.5
**Quem dá suporte após a alta**		
Amigo/vizinho	1	2.5
Filhos	11	27.5
Cônjuge/companheiro(a)	24	60.0
Irmão(ã)	4	10.0

**Tabela 2 -:** Distribuição numérica e porcentual dos participantes, segundo as variáveis clínicas (n = 40). Vila Nova de Gaia, VNG, Portugal, 2022-2023

Variáveis	Participantes (n)	Percentual (%)
**Histórico clínico**		
Doença arterial periférica	40	100.0
Dislipidemia	2	5.0
Hipertensão	20	50.0
Fumante	7	17.5
Doença pulmonar obstrutiva crônica	7	17.5
Doença cardíaca	7	17.5
Insuficiência renal	7	17.5
Diabetes	18	45.0
Retinopatia diabética	1	2.5
Anemia crônica	1	2.5
Insuficiência respiratória	1	2.5
Bexiga hiperativa	1	2.5
Artrite reumatoide	1	2.5
**Total de comorbidades**		
Uma comorbidade	5	12.5
Duas comorbidades	13	32.5
Três comorbidades	13	32.5
Quatro comorbidades	6	15.0
Cinco comorbidades	3	7.5
**Nível de amputação**		
Amputação transfemoral	31	77.5
Amputação transtibial	9	22.5
**Amputação contralateral anterior**		
Ausente	34	85.0
Amputação de dedo	3	7.5
Amputação transmetatársica	3	7.5
**Há quanto tempo a amputação foi feita?**		
< 1 ano	6	15.0
1-5 anos	20	50.0
> 5 anos	14	35.0
**Dispositivos de assistência para mobilidade**		
Cadeira de rodas (apenas)	29	72.5
Cadeira de rodas e muletas	2	5.0
Prótese e cadeira de rodas	4	10.0
Prótese e muletas	3	7.5
Prótese (apenas)	2	5.0

* n = Número de participantes

### Grau de dependência por domínio de autocuidado

A avaliação da dependência da AMI no autocuidado foi realizada de acordo com os 10 domínios incluídos na versão curta do SCDEF, ou seja, “andar”, “transferir-se”, “virar-se”, “levantar”, “usar o banheiro”, “alimentar-se”, “arrumar-se”, “vestir-se e despir-se”, “tomar banho” e “tomar medicamento”. O autocuidado é classificado por meio de uma escala Likert de quatro pontos em cada domínio de autocuidado: (1) dependente não participa, (2) precisa de ajuda de outra pessoa, (3) precisa de dispositivos de assistência e (4) totalmente independente, o que ajuda a avaliar o nível de dependência e as necessidades do amputado de MI em relação às atividades da vida diária.

Em relação ao autocuidado “andar”, três atividades foram incluídas nesse domínio para avaliar o autocuidado, ou seja, manter o corpo em uma posição ereta, ficar de pé sem qualquer tipo de ajuda de dispositivos de assistência ou de outra pessoa, usar dispositivos de assistência que incluem muletas ou prótese. Quanto aos três domínios incluídos, 5% dos amputados de MI conseguem ficar de pé sem qualquer tipo de ajuda de dispositivos de assistência ou de outra pessoa, 70% precisam usar dispositivos de assistência que incluem muletas ou prótese e 12,5% não conseguem ficar de pé, respectivamente.

Com relação à atividade de subir e descer escadas, 70% dos amputados de MI são incapazes de fazê-lo, 22,5% sobem e descem escadas usando muletas e prótese e 7,5% podem subir e descer escadas com a ajuda de uma pessoa. Na atividade de caminhar distâncias médias, 72,5% dos amputados de MI conseguem fazê-lo com a ajuda de muletas, prótese ou cadeira de rodas; 7,5% dos participantes não conseguiram caminhar distâncias, mesmo com ajuda ou dispositivos de assistência.

No autocuidado “Transferir-se”, que avalia a capacidade dos amputados de MI de se transferirem da cama para a cadeira/cadeira de braços e da cadeira/cadeira de braços para a cama, 62,5% exigem o uso de dispositivos de assistência para realizar a transferência, 27,5% não conseguem realizar a transferência de forma independente ou com o uso de dispositivos de assistência e exigem a ajuda de outra pessoa para realizar a transferência. Apenas 10% dos amputados de MI conseguiram transferir-se da cama para a cadeira/cadeira de braços e da cadeira para a cadeira de braços de forma independente, sem o uso de dispositivos de assistência ou ajuda de outra pessoa.

Em relação ao domínio de autocuidado “virar-se”, o qual avalia se a pessoa com amputação de membro inferior move o corpo de um lado para o outro, os resultados mostram que 65% dos amputados precisam de dispositivos de assistência, 7,5% conseguem se virar somente com a ajuda de outra pessoa e 27,5% são totalmente independentes nesse domínio. No que se refere ao autocuidado “levantar”, que consiste na capacidade de levantar uma parte do corpo, a maioria dos participantes, 52,5% dos amputados, é independente, 40,0% precisam de dispositivos de assistência e 7,5% precisam da ajuda de outra pessoa.

No autocuidado “usar o banheiro”, em relação à atividade de posição no vaso sanitário ou na arrastadeira, 77,5% dos amputados de MI precisam de dispositivos de assistência; na habilidade de levantar-se do vaso sanitário, 72,5% dos participantes precisam de dispositivos de assistência; em relação à atividade de arrumar as roupas após a higiene pessoal, 27,5% precisam de dispositivos de assistência e 45% são completamente independentes.

Em relação ao autocuidado “alimentar-se”, a maioria dos amputados de MI (92,5%) são independentes para abertura dos recipientes, 97,5% são independentes para segurar um copo ou uma xícara, mas 87,5% precisam da ajuda de outra pessoa para preparar alimentos para comer.

O cuidado com as unhas é a atividade relacionada ao autocuidado “arrumar-se” com maior dependência, com 97,5% precisando da ajuda de outra pessoa; em relação às atividades pentear o cabelo, aplicar desodorante e manter a higiene bucal, a maioria dos participantes é independente.

O autocuidado “vestir-se e despir-se” inclui cinco atividades; nelas, 67,5% dos amputados de MI precisam da ajuda de outra pessoa para amarrar os sapatos e 65% precisam da ajuda de outra pessoa para calçar as meias. Um total de 82,5% eram totalmente independentes para se vestir, 52,5% precisavam da ajuda de outra pessoa para escolher roupas e 47,5% precisavam da ajuda de outra pessoa para vestir a parte inferior do corpo.

Uma das atividades de autocuidado com maior nível de dependência na pessoa com AMI é a atividade de autocuidado “tomar banho”, relacionada à obtenção de itens para o banho; 92,5% dos amputados de MI precisam da ajuda de outra pessoa, na capacidade de lavar o corpo, um total de 85,0% precisa da ajuda de outra pessoa. Um total de 55% dos amputados de MI eram completamente independentes na abertura da torneira para o banho. Com relação à atividade de autocuidado “tomar remédio”, 85,0% dos amputados de MI precisam da ajuda de outra pessoa para preparar a medicação, e com relação à atividade “tomar remédio”, 82,5% são independentes.

 Para criar uma variável geral, os itens de cada domínio foram combinados. A Tabela 3 mostra a conversão das dez variáveis calculadas relacionadas a diferentes atividades de autocuidado em uma única variável composta chamada de nível de dependência geral. 

 A Tabela 3 mostra que o domínio de autocuidado com alto nível de dependência é o autocuidado “andar”, com média de 2,28 e desvio-padrão de 0,59. A seguir encontrou-se o autocuidado “tomar banho”, com média de 2,41 e desvio-padrão de 0,47; autocuidado “vestir-se e despir-se” com média de 2,81 e desvio-padrão de 0,61%; autocuidado “usar o banheiro” com média de 2,83 e desvio-padrão de 0,65; e autocuidado “transferir-se” com média de 2,83 e desvio-padrão de 0,56%. 

**Tabela 3 -:** Média, desvio-padrão, mínimo e máximo para cada domínio do autocuidado. Vila Nova de Gaia, VNG, Portugal, 2022-2023

Domínios do autocuidado	n*	Mínimo	Máximo	Média	Desvio-padrão
Andar	40	1.00	3.33	2.28	0.59
Transferir-se	40	2.00	4.00	2.83	0.59
Virar-se	40	2.00	4.00	3.20	0.56
Levantar	40	2.00	4.00	3.45	0.64
Usar o banheiro	40	1.00	3.33	2.83	0.65
Alimentar-se	40	1.75	4.00	3.45	0.32
Arrumar-se	40	1.25	3.50	3.37	0.48
Vestir-se e despir-se	40	1.20	3.80	2.81	0.61
Tomar banho	40	1.00	3.33	2.41	0.47
Tomar medicamentos	40	1.50	4.00	3.19	0.75
Nível geral de dependência	40	1.38	3.52	2.99	0.43

* n = Número de participantes do estudo

Ao considerar os domínios de autocuidado com menores níveis de dependência, o autocuidado “alimentar-se” mostrou-se com o menor nível de dependência, com média de 3,45% e desvio-padrão de 0,32%, seguido pelo autocuidado “levantar” com média de 3,45% e desvio-padrão de 0,64%; autocuidado “vestir” com média de 3,37% e desvio-padrão de 0,48%; autocuidado “virar-se” com média de 3,20% e desvio-padrão de 0,56%; e autocuidado “tomar medicamentos” com média de 3,19% e desvio-padrão de 0,75%.

O nível global de dependência de autocuidado dos amputados de MI, neste estudo, apresentou média de 2,99% com desvio-padrão de 0,43%, e quase todos os participantes necessitaram de assistência nos domínios de autocuidado avaliados. Os tipos de dispositivos de assistência usados por amputados de MI para auxiliar nas atividades de autocuidado incluíam dispositivos ambulatoriais, como cadeiras de rodas, muletas e prótese. Os amputados de MI usavam bancos de banho, barras de apoio e cadeiras de banho como dispositivos de assistência para ajudar no banheiro. No quarto, eles usavam a cabeceira e as bordas da cama para auxiliar na virada, elevação e transferência, e não usavam dispositivo de assistência para se adaptar à cama e auxiliar nas atividades de autocuidado.

 A análise de *cluster* em duas etapas identificou dois *clusters* distintos dentro da amostra de indivíduos com amputação maior de membro inferior maior de causa vascular, com altas pontuações de silhueta indicando boa coesão e separação entre os *clusters* . O *cluster* 1 incluiu uma proporção maior de pacientes mais velhos, frágeis e dependentes. A Tabela 4 fornece uma descrição detalhada dos *clusters* formados, incluindo suas características sociodemográficas e clínicas, bem como seu nível de dependência no autocuidado. 

 Maior autonomia no uso do banheiro, deambulação e transferência da cama para a cadeira mostraram-se atividades de autocuidado com melhor capacidade de predizer a autonomia desse tipo de paciente ( Figura 1 ). 

**Tabela 4 -:** Características do *cluster* . Vila Nova de Gaia, VNG, Portugal, 2023

	Cluster 1 (n*=9)	Cluster 2 (n*=31)
Masculino	88.8% (8)	77.4% (24)
Escolaridade primária	100% (9)	61.3% (19
Amputação transfemoral	88.8% (8)	74.2% (23)
Andar	M=1.44	M=2.53
Transferir-se	M=2.00	M=3.06
Usar o banheiro	M=1.85	M=3.11
Alimentar-se	M=3.22	M=3.52
Arranjar-se	M=2.92	M=3.50
Vestir-se e despir-se	M=2.09	M=3.01
Levantar	M=2.78	M=3.65
Virar-se	M=2.67	M=3.35

* n = Número de participantes

## Discussão

Este estudo é o primeiro a examinar o nível de dependência de autocuidado nas atividades de vida diária, em indivíduos que sofreram uma amputação de membro inferior maior de origem vascular. Para este estudo utilizou-se um instrumento que permite uma avaliação priorizada e operacional das competências de autocuidado em indivíduos com amputações maiores de membros inferiores, divididas em 10 domínios.

 Ao analisar os indivíduos que sofreram uma amputação maior de membro inferior de causa vascular obteve-se, além de informações valiosas sobre as suas características sociodemográficas e clínicas, o nível de dependência de assistência para as atividades da vida diária. Em relação ao sexo, 40 (80%) dos participantes eram homens, e 20%, mulheres. Estudos anteriores mostram que os homens têm um risco maior de amputação de membros inferiores do que as mulheres ^(^ 19 ^)^ , o que pode ser explicado por taxas mais altas de doença arterial periférica, neuropatia periférica e tabagismo em homens do que em mulheres ^(^ 20 ^-^ 21 ^)^ . A menor incidência de amputação de membros inferiores em mulheres com doença vascular pode ser explicada pelo efeito do estrogênio na redução da patologia vascular ^(^ 22 ^)^ . 

 Quanto à idade dos amputados de membros inferiores, a faixa etária mais frequente foi entre 76 e 80 anos, representando 27,5% da amostra, e a faixa etária menos comum foi entre 46 e 50 anos, representando apenas 2,5% dos participantes. Evidências anteriores sugerem que a maioria das amputações atuais é secundária a doenças de causa vascular e a prevalência de DAP aumenta significativamente com a idade, tanto em homens quanto em mulheres. Em pessoas com idade inferior a 50 anos, a prevalência é de 15% a 20%, aumentando para 15% a 20% aos 80 anos de idade ^(^ 23 ^-^ 24 ^)^ . 

**Figura 1:** - Preditores de cluster

 No presente estudo, 40% dos amputados de membros inferiores com disfunção vascular encontravam-se aposentados precocemente antes da amputação. Estudos anteriores relataram que a claudicação intermitente é uma manifestação de doença arterial periférica, resultando em mobilidade e qualidade de vida reduzidas ^(^ 25 ^)^ . Mesmo em pacientes com doença arterial periférica com ou sem sintomas atípicos, o comprometimento funcional está presente; a fragilidade é comum em pacientes com DAP sintomática e está associada ao comprometimento da marcha ^(^ 25 ^-^ 26 ^)^ . 

 Os pacientes mais velhos têm menor probabilidade de locomoção com prótese do que os mais jovens. Isso ocorre devido ao nível de amputação dos pacientes com amputação transtibial, pois eles têm duas vezes mais probabilidade de locomoção com prótese do que os pacientes com amputação transfemoral ^(^ 27 ^)^ . Na amostra do estudo, 72,5% dos amputados de membros inferiores tinham idade superior a 65 anos de idade, 77,5% tinham amputação transfemoral e 72,5% estavam confinados a uma cadeira de rodas. Pesquisadores ^(^ 28 ^)^ relataram que, em um período de três meses após a amputação, todos os grupos etários de amputados de membros inferiores apresentaram pontuações mais baixas para as funções físicas e que, após 12 meses de amputação, há uma diferença entre os grupos etários em nível funcional, com uma perda óbvia de função nos pacientes mais velhos. Esses pacientes precisam de um foco especial que requer reabilitação diária para recuperar suas funções físicas básicas. A função física em amputados de membros inferiores também pode ser afetada pelo número de comorbidades presentes. 

 Nossos resultados mostraram que 65% dos amputados de membros inferiores tinham pelo menos duas ou três comorbidades. Adultos que sofreram amputação de membros inferiores devido a doenças vasculares geralmente têm vários problemas de saúde ^(^ 29 ^)^ . A presença de múltiplas comorbidades em pacientes com doença vascular periférica avançada geralmente resulta em níveis elevados de fragilidade ^(^ 30 ^)^ . 

 Resultados da função física ruim, incluindo problemas de equilíbrio, diminuição da força e mobilidade limitada ou capacidade de andar, são comumente observados em pacientes com amputações de membros inferiores de causa vascular. As pessoas com amputações de membros inferiores de causa vascular geralmente apresentam uma série de problemas de saúde que afetam seu bem-estar físico e sua capacidade de realizar tarefas diárias ^(^ 10 ^,^ 31 ^)^ . Nosso estudo constatou que, estatisticamente, os domínios de autocuidado com maior dependência em amputados de membros inferiores maiores com disfunção vascular foram “caminhar”, “tomar banho”, “vestir-se e despir-se”, “usar o banheiro”, “transferir-se”, sendo o autocuidado de caminhar o de maior dependência em nossos participantes. Caminhar e subir escadas são as atividades mais difíceis para indivíduos que sofreram amputação de membros inferiores. Os indivíduos com amputações transfemorais são particularmente afetados e não conseguem recuperar a capacidade de realizar atividades básicas e instrumentais da vida diária ^(^ 32 ^)^ . 

 Em relação às áreas de autocuidado com maior dependência de AVDs identificadas em nosso estudo, a literatura indica que os pacientes com mais de 12 meses após a amputação precisam de melhor assistência com higiene pessoal, tomar banho, vestir-se e despir-se e ir ao banheiro ^(^ 28 ^)^ . O declínio mais significativo na mobilidade, particularmente na marcha independente, foi observado 12 meses após a amputação. A capacidade do paciente de andar sem ajuda depende da presença de um membro protético ^(^ 28 ^)^ . De todos os participantes de nosso estudo, apenas 5% conseguiram andar usando apenas prótese. Doze meses após a amputação, a maioria dos amputados de membros inferiores ainda não consegue recuperar o mesmo nível de independência em suas atividades diárias ^(^ 28 ^)^ . 

 Os enfermeiros desempenham um papel importante no incentivo aos amputados de membros inferiores com disfunção vascular para que recuperem a independência nas atividades de autocuidado. De acordo com nossa análise de agrupamento, uma maior autonomia de autocuidado no uso do banheiro, na caminhada e na transferência da cama para a cadeira pode prever a autonomia nesse tipo de paciente. Estudos anteriores mostraram que a caminhada ajuda na recuperação da independência na realização de habilidades básicas e atividades da vida diária ^(^ 33 ^)^ . Intervenções de reabilitação domiciliar para amputados de membros inferiores de causa vascular com exercícios para melhorar o equilíbrio em pé, a capacidade de sentar e ficar em pé, as transferências e a mobilidade ajudam esses indivíduos a recuperar a mobilidade e a independência funcional mais cedo, contribuindo para melhorar a mobilidade e a qualidade de vida ^(^ 34 ^)^ . 

Este estudo tem algumas limitações, como o tamanho da amostra não probabilística de pacientes de um único hospital no norte de Portugal. Portanto, deve-se ter cautela ao generalizar os resultados. Para aprimorar estudos futuros, recomenda-se aumentar o tamanho da amostra e estender a duração do estudo. Apesar das limitações, este estudo lança luz sobre o impacto da amputação de membros inferiores com disfunção vascular grave nas atividades da vida diária. Essas descobertas podem fornecer subsídios para o planejamento de pesquisas futuras sobre a dependência de uma pessoa com amputação maior de membro inferior de causa vascular em atividades da vida diária e para o desenvolvimento de intervenções e programas para ajudar no treinamento de pacientes, cuidadores e familiares.

## Conclusão

 Este estudo mostrou que o domínio de autocuidado com maior nível de dependência é *“andar”,* e o menor *, “alimentar-se”.* Maior autonomia no uso do banheiro, deambulação e transferência da cama para a cadeira mostraram-se atividades de autocuidado com melhor capacidade de predizer a autonomia de indivíduos com amputação de causa vascular. Os indivíduos com esse tipo de amputação têm idade superior a 65 anos de idade; apenas 5% são capazes de deambular com prótese e têm algum nível de dependência nos domínios de autocuidado relacionados às atividades da vida diária. 

Uma ferramenta de avaliação confiável para avaliar o nível de dependência nos diferentes domínios de autocuidado de uma pessoa com amputação maior de membro inferior de causa vascular, ajuda a identificar as necessidades em relação às atividades de autocuidado. Identificar as necessidades pode contribuir para a compreensão da limitação funcional e incapacidade de uma amputação de causa vascular e o suporte de que o paciente necessita para realizar as suas atividades diárias.

Atualmente, há uma tendência de alta precoce com retorno antecipado à comunidade/casa quando o amputado ainda é totalmente dependente de outras pessoas. Nesse sentido, é essencial avaliar a dependência de uma pessoa com uma amputação maior de membro inferior de causa vascular nas atividades da vida diária, para, dessa forma, projetar intervenções e programas educacionais e melhorar sua capacidade de realizar atividades básicas da vida diária. As intervenções de reabilitação devem continuar após a alta com exercícios para melhorar a capacidade funcional e a independência nas atividades de autocuidado. Os cuidadores familiares devem estar envolvidos desde o início dessas intervenções e programas para apoiar o paciente que foi submetido a amputação de membro inferior a recuperar algum nível de independência na realização de atividades da vida diária, contribuindo assim para a sua autonomia.
